# Seeds Priming with Bio-Silver Nanoparticles Protects Pea (*Pisum sativum* L.) Seedlings Against Selected Fungal Pathogens

**DOI:** 10.3390/ijms252111402

**Published:** 2024-10-23

**Authors:** Karolina Stałanowska, Viorica Railean, Paweł Pomastowski, Agnieszka Pszczółkowska, Adam Okorski, Lesław Bernard Lahuta

**Affiliations:** 1 Department of Plant Physiology, Genetics and Biotechnology, University of Warmia and Mazury in Olsztyn, Oczapowskiego 1A, 10-719 Olsztyn, Poland; karolina.stalanowska@uwm.edu.pl; 2 Department of Infectious, Invasive Diseases and Veterinary Administration, Institute of Veterinary Medicine, Nicolaus Copernicus University in Toruń, Gagarina 7, 87-100 Toruń, Poland; viorica.railean@umk.pl; 3 Centre for Modern Interdisciplinary Technologies, Nicolaus Copernicus University in Toruń, Wileńska 4, 87-100 Toruń, Poland; p.pomastowski@umk.pl; 4 Department of Inorganic and Coordination Chemistry, Nicolaus Copernicus University in Toruń, Gagarina 7, 87-100 Toruń, Poland; 5 Department of Entomology, Phytopathology and Molecular Diagnostics, University of Warmia and Mazury in Olsztyn, Pl. Łódzki 5, 10-727 Olsztyn, Poland; agnieszka.pszczolkowska@uwm.edu.pl (A.P.); adam.okorski@uwm.edu.pl (A.O.)

**Keywords:** *Pisum sativum*, seed, nano-priming, silver, fungal pathogens

## Abstract

Nano-priming is a relatively new seed treatment technique using metal and metal oxide nanoparticles (NPs), and such application of NPs may support the plants’ immunity. Recently we have shown that the that biologically synthesized silver nanoparticles (bio-AgNPs) used as short-term foliar treatment protect pea seedlings against *D. pinodes* and *F. avenaceum*. In the present study, the protection of peas against both fungal pathogens via seed priming with bio-AgNPs was analyzed. Moreover, the changes in the polar metabolic profiles of the seedlings caused by priming and infection were also compared. Seed priming with bio-AgNPs at concentrations of 50 and 100 mg/L considerably reduced the symptoms and infection levels of both pathogens by over 70% and 90% for *F. avenaceum* and *D. pinodes*, respectively. Pathogens infection and nano-priming affected the metabolic profile of pea seedlings. The major changes in the primary metabolism were observed among carbohydrates and amino acids. In turn, this may result in changes in the expression and accumulation of secondary metabolites. Therefore, further investigation of the effect of nano-priming should focus on the changes in the secondary metabolism.

## 1. Introduction

Seed priming, also called seed coating or dressing, is a well-known agricultural technique of seed pre-sowing treatment. In this procedure, dry seeds are soaked in water or water solutions of salts, osmolytes, or phytohormones for a restricted time (without radical protrusion) and then dried to their original moisture content. During seed imbibition, the metabolism in the embryo (and storage tissues) is activated, while the drying process induces protective pathways [1,2,3]. Seed priming positively affects followed water uptake (during germination), triggers the antioxidative system, DNA reparation, synthesis of stress-related proteins, and mobilization of storage materials. Moreover, seed priming improves and synchronizes seed germination and vigor. Faster germination contributes to faster seedling growth and plant development, reducing the time of seedling exposure to unfavorable environmental conditions after germination, which is crucial for successful crop establishment [3,4,5].

The seed priming process can cause moderate stress to the seed which induces stress/priming memory that may increase resistance to other stress factors [3,4,5]. Conditions such as duration of priming, time and temperature of subsequent seeds drying, agents, and their concentrations used for priming are major factors that should be considered and adjusted for effective seed priming of particular plant species [2,4]. We can distinguish many types of priming, depending on the agents used for seed priming, like hydro/water-priming, osmo-priming, halopriming, and bio-priming using beneficial microorganisms [1,2].

A relatively new priming technique is nano-priming, which exploits nanomaterials such as metal or metal-oxide nanoparticles (NPs) [6,7]. During seed imbibition in suspensions of metalloid NPs and followed seed drying, NPs can penetrate the seed coat and reach embryonic tissues, but most of them remain on the seed surface [8,9,10,11]. Moreover, the positive or negative effects of NPs on plants depend not only on the physicochemical properties and concentration of NPs but also on the plant species. Therefore, nano-priming should be properly optimized to utilize only their benefits for crops [12,13].

Nano-priming enhanced water uptake by imbibed dry seeds, presumably via the creation of nanopores or upregulation of aquaporin gene expression. Moreover, NPs trigger the generation of reactive oxygen species (ROS) as well as antioxidative systems. In turn, ROS causing loosening of the cell walls of the seed coat may facilitate the penetration of NPs into the seed and activate starch hydrolysis, resulting in accelerated germination and increased seedling’s vigor. Increased gene expression of phenylalanine ammonia-lyase (PAL) and pathogenesis-related proteins (PR-proteins) was also observed. Additionally, NPs improve the innate immune system of plants, which increases/enhances their tolerance to abiotic and biotic stresses [3,7,14].

Regarding the strong antibacterial and antifungal properties of metalloid NPs, they could be implemented for plant protection as pesticides, even more effective than traditional ones [3,12,13]. Additionally, seed nano-priming appears to be more effective and economically accurate than foliar sprays in protecting plants against fungal phytopathogens, infecting plants at an early stage of development, or causing soil-borne diseases [15]. ZnO NPs priming of chickpea seeds successfully reduced Fusarium wilt caused by *Fusarium oxysporum* [16]. Tomato plants were more resistant to *Phytopthora infestans* after SeNPs seed priming [17]. AgNPs successfully reduced wheat kernels infestation by *Aspergillus niger* and *A. flavus* [18]. Kashyap and Siddiqui [19,20] showed that ZnO NPs and SiO_2_ NPs priming more efficiently reduced infection of peas by *Meloidogyne incognita* (nematode) and *Pseudomonas syringae* pv. *pisi* (bacteria) than foliar spray. However, no study has employed nano-priming against *Fusarium avenaceum* or *Didymella pinodes* infection, causing pea root rot and Ascochyta blight, respectively, a dangerous fungal disease in peas, an important legume crop [21,22].

In our recent publication, we demonstrated that biologically synthesized silver nanoparticles (bio-AgNPs), produced using *Lactobacillus paracasei*, showed antifungal properties. In the experiment, short-term immersion of few-day-old pea seedlings in bio-AgNPs suspensions enabled the successful reduction of infection of pea by *Didymella pinodes* and *Fusarium avenaceum* [23]. Therefore, this study aimed to investigate whether using bio-AgNPs for pea seed priming could also limit the infection of these phytopathogens. Moreover, the changes in the polar metabolic profiles of infected and uninfected seedlings developed from nano-primed seeds were compared.

## 2. Results and Discussion

### 2.1. Preliminary Studies on Seeds Priming

The optimization seeds priming process included testing the effects of the duration of seed imbibition (for 0.5–4 h) in the water before followed seeds drying (in laboratory conditions for one week), on the seed germinability and seedling growth. Although this water-priming had no negative effect on seeds germinability, it strongly decreased seedling’s growth, when seeds were imbibed for 2 or 4 h (Table S1). For roots, this negative effect was observed for more than 2 h of priming; whereas for shoots, it was observed for 4 h of priming. Results of other studies, focusing on the optimization of pea seeds hydropriming, also indicated that a short time of seed soaking (below 1 h) is safe for seed germination and further seedling development [24]. The negative effect of longer imbibition may be related to mitochondrial damage [25]. However, the rate of embryonic tissue rehydration during seed soaking or germination, as well as the rate of followed seed drying, seem to be the most important factors affecting embryo viability and vigor due to the loss of membrane integrity and oxidative damages [26,27,28].

In the second experiment, the pea seeds were imbibed in the water suspension of bio-AgNPs (at a concentration of 50–200 mg/L), for 0.5 to 1.5 h and then left to dry (as above). This priming had no negative effect on seed germinability. Moreover, the length of the root and epicotyl of 4-day-old seedlings (developed from nano-primed seeds) did not differ between the priming times within each nanoparticle concentration. However, both factors had a significant (*p* ≤ 0.05) negative effect on the seedlings’ length compared with seedlings developed from water-primed seeds (Table 1). This was especially noticeable for epicotyls developed from seeds primed with bio-AgNPs at 100–200 mg/L. Priming of pea seeds with bio-AgNPs at 50 mg/L for 1 and 1.5 h and priming with bio-AgNPs at higher concentrations negatively affected also the fresh and dry weight (FW and DW, respectively) of the roots, presumably due to absorption of higher amounts of AgNPs by primed seeds (Table 1).

However, in the present study, the concentration of Ag in seedling tissues was not analyzed. The exact mechanism of nanoparticle absorption, translocation, and accumulation during nano-priming remains unknown [14]. Part of the nanoparticles stay on the seed surface and can penetrate some layers of the seed coat [29], but some can enter the seed through nano-scale pores in the seed coat, reaching the embryo [8,9,10,11]. Such diffusion through pores in the cell wall and cell membrane may be considered only for small nanoparticles as the size exclusion limit of plant cell walls ranges from 5 to 20 nm. Larger nanoparticles that remain at the seed coat can release silver ions or biomolecules that could cross the cell wall [30]. It is also known that nanoparticles can enter plant cells via endocytosis (fluid phase or clathrin-dependent) [14,30]. Moreover, the participation of aquaporins [31] and ion channels in nanoparticle transport is possible [30]. Additionally, it is suggested that the loosening of cell wall structure (caused by ROS) may contribute to the penetration of NPs into the seed [14], enhancing enzyme activity and regulating hormone levels, leading to increasing seed vigor and stress tolerance [7,14,31,32]. When nanoparticles enter the embryo, they can be transported in tissues through apoplastic or symplastic pathways [14,30]. Thus, the analysis focusing on the dynamics of silver nanoparticles absorption and possible their conversion into Ag^+^ in primed/germinating pea seeds and developing seedling tissues requires separate experiments, in the future.

Nonetheless, based on the results of the preliminary experiments, described above, seed priming with bio-AgNPs at concentrations of 50 and 100 mg/L for 1 h was chosen to test pea seedlings’ resistance against fungal infection.

### 2.2. Seed Priming with Bio-AgNPs Improves Seedlings Resistance Against Fungal Pathogens

The uninfected 21-day-old seedlings that developed from seeds primed with bio-AgNPs had no negative symptoms compared to the control (seedlings developed from water-primed seeds, Figures S1–S3). No significant differences (*p* ≤ 0.05) were observed in the shoot’s length, FW of the shoot, FW of roots, and seedling DW (Table S2). Moreover, seedlings from seeds primed with 100 mg/L bio-AgNPs showed increased primary root length (Table S2).

**Figure 1:**
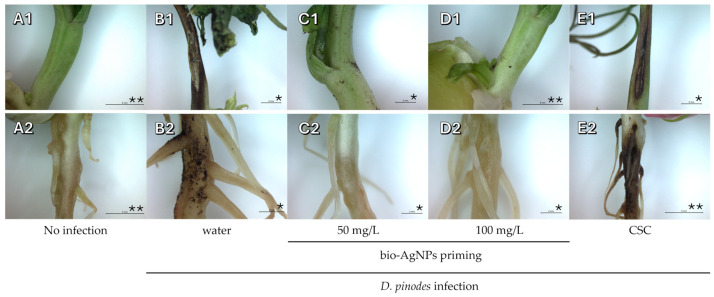
The base of shoot and primary root (**A1**–**E1** and **A2**–**E2**, respectively) of 21-day-old uninfected seedlings of pea (developed from seeds primed with water, **A1** and **A2**) and seedlings infected with *D. pinodes* (**B**–**E**). The infected seedlings developed from seeds primed with water (**B1**,**B2**), bio-AgNPs at concentrations of 50 mg/L (**C1**,**C2**) or 100 mg/L (**D1**,**D2**), and a commercial seed coating (CSC) mixture (Maxim 025 FS with 2.38% fludioxonil 25 g/L, **E1**,**E2**). Scale bars: * = 2 mm and ** = 5 mm.

The inoculation of 7-day-old seedlings developed from water- and commercial seed coating (CSC) mixture-primed seeds with *D. pinodes* resulted in strong development of infection in both shoots and roots during the next 14 days of seedlings growth (Figure 1). However, in seedlings developed from bio-AgNPs-primed seeds (at both concentrations), only a few infection symptoms were observed (mostly in the region of fungal inoculation, Figure 1C,D and Figure S2).

**Figure 2:**
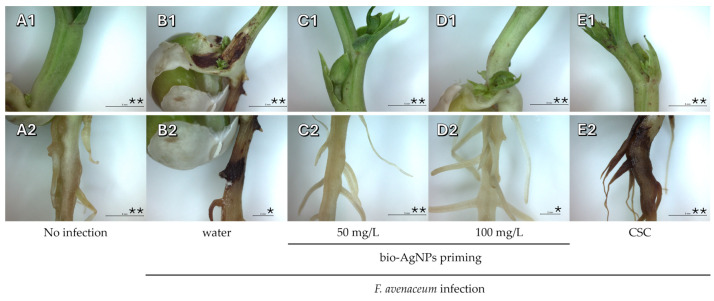
The base of shoot and primary root (**A1**–**E1** and **A2**–**E2**, respectively) of 21-day-old uninfected seedlings of pea (developed from seeds primed with water, **A1**,**A2**) and seedlings infected with *F. avenaceum* (**B**–**E**). The infected seedlings developed from seeds primed with water (**B1**,**B2**), bio-AgNPs at concentrations of 50 mg/L (**C1**,**C2**) or 100 mg/L (**D1**,**D2**), and a commercial seed coating (CSC) mixture (Maxim 025 FS with 2.38% fludioxonil 25 g/L, **E1**,**E2**). Scale bars: * = 2 mm and ** = 5 mm.

Similarly, seeds priming with bio-AgNPs protected developing seedlings against infection of roots by *F. avenaceum*, whereas clear signs of infection were visible in the case of inoculated seedlings grown from water- and CSC-primed seeds (Figure 2 and Figure S3). Observed symptoms were typical for those pathogens. Both are associated with pea root rot which appears as dark-brow lesions and spots on roots, base of the shoots, and cotyledons, and in more mature plants also leaf yellowing [33,34]. *D. pinodes* is also the most damaging and pathogenic causal agent of Ascochyta blight, which causes dark-brownish necrotic lesions on the aboveground parts of plants and the base of the roots [35].

**Figure 3:**
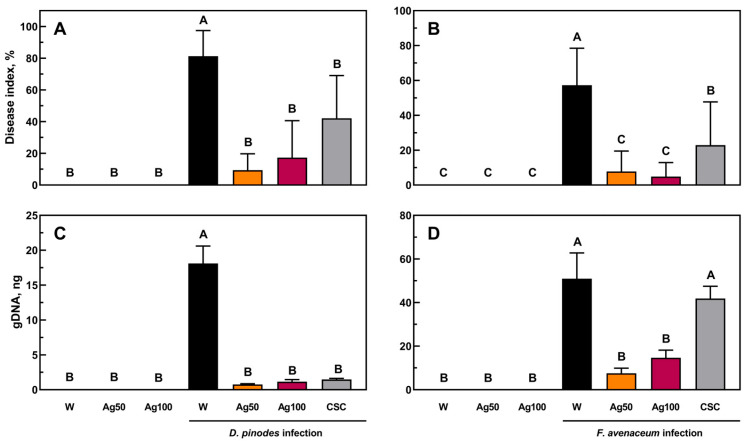
The disease index (the percentage of disease symptoms) of pea seedlings grown from seeds primed with water (W, black), bio-AgNPs at concentrations of 50 mg/L (Ag50, orange) and 100 mg/L (Ag100, burgundy), and a commercial seed coat (CSC, grey), caused by *D. pinodes* (**A**) and *F. avenaceum* (**B**), and the quantity of *D. pinodes* (**C**) and *F. avenaceum* (**D**) measured by qPCR in the pea seedlings 14 days post-inoculation. Values are the means of 3 replicates + SD. The same letters above the bars indicate statistically insignificant (*p* ≤ 0.01) differences based on two-way ANOVA and Tukey’s post-hoc test.

The inhibitory effect of seed nano-priming on *D. pinodes* and *F. avenaceum* infections was also confirmed by a lower disease index (Figure 3A,B) and lower content of pathogens gDNA in seedling tissues, as confirmed by qPCR (Figure 3C,D). Moreover, the disease index and the content of the *D. pinodes* gDNA in seedlings developed from bio-AgNPs-primed seeds were comparable to those found in seedlings from CSC-primed seeds (Figure 3A,C). In the case of *F. avenaceum* inoculation, bio-AgNP priming at both concentrations reduced the symptoms and level of infection more than CSC did (Figure 3B,D).

Thus, the present study confirms our last suggestion that bio-AgNPs can be successfully used for pea protection against *D. pinodes* and *F. avenaceum*, not only by seedling foliar treatment [23] but also via seed priming. The foliar treatment decreased the level of infection of *D. pinodes* by 91 to 98% and disease index by 91 to 94% at concentrations of 100 and 200 mg/L, respectively [23], whereas seed nano-priming (in the present study) reduced the level of infection by 93 to 95% (Figure 3A) and disease index by 88 to 78% (Figure 3C, at concentrations of 50 and 100 mg/L, respectively).

In the case of *F. avenaceum*, the gDNA level was decreased by over 71% by both seed priming (Figure 3D) and foliar treatment [23], and the DI decreased by 86–92% after seed priming (Figure 3B) and 75–85% after foliar treatment [23]. The fungicidal efficacy of bio-AgNPs is probably due to their high metallic silver content and specific coating, which may affect the stability and bioactivity of the nanoparticles, as previously suggested [23,36].

The nano-priming with AgNPs can suppress infection of selected soilborne pathogens and also of other legumes-common bean (*Macrophomina phaseolina*, *Pythium graminicola*, *Rhizoctonia solani*, and *Sclerotium rolfsii*) [37] and chickpea (*F. oxysporum* f.sp. *ciceri*) [38]. On the other hand, the foliar application of AgNPs (100 and 200 mg/L) against selected bacteria (*Pseudomonas syringae* pv. *tomato*, *Xanthomonas campestris* pv. *vesicatoria*, *Pectobacterium carotovorum*, subsp. *carotovorum,* and *Ralstonia solanacearum*) and fungi (*Fusarium oxysporum* f.sp. *lycopersici* and *Alternaria solani*) were more successful in reducing tomato plant infection than seed priming [39]. Thus, both methods of AgNPs application can be used, regarding plant species and types of AgNPs. Moreover, bio-AgNPs (the same as used in the present study) exhibited no inhibitory effect on strains *Lactococcus lactis* and *Lactobacillus casei*, but they strongly inhibited the growth of other microorganisms, i.e., for *Saccharomyces cerevisiae* (MIC = 3.12 µg/mL), *Proteus mirabilis*, *Acinetobacter baumannii*, *Pseudomonas aeruginosa*, *Staphylococcus epidermidis,* and *Micrococcus luteus* (MIC = 1.56 µg/mL for all mentioned bacteria) [36]. It should also be noted that the antibacterial properties of biologically synthesized silver nanoparticles can be regulated by nanoparticle functionalization with antibiotics [40].

Moreover, the toxicity of AgNPs was also presented to other organisms, such as plants, soil microbial communities, nematodes, insects, and aquatic organisms (like *Daphnia* spp. or zebrafish). Therefore, nanoparticle application can lead to the contamination of the environment [41,42]. The negative effects of AgNPs, besides the dosage used for application, depend on their physicochemical characteristics, including their size, shape, surface coating, and charge and aggregation ability, which affects their stability and reactivity [41,42]. Indeed, in our previous studies bio-AgNPs (in vitro conditions) showed an inhibitory effect on wheat seedlings’ growth and development [43]. In contrast, similar low concentrations of chemically synthesized AgNPs and silver ions (applied as AgNO_3_) stimulated pea seed germination and seedling growth. Negative effects of Ag^+^ were observed after pea seeds 8 h imbibition in AgNO_3_ at concentrations 500 and 1000 mg/L [44]. Other researchers also suggest that AgNP toxicity to plants is species-specific [42,45].

The sowing of nano-primed seeds can lead to contamination of soil, which is a very complex matrix [41,42]. In epigeally germinating pea seeds, cotyledons (with seed coat) remain in the soil. Therefore, it could be expected that the AgNPs presented the seed’s coat surface as well as those absorbed by cotyledons that remain in the soil, affecting soil microbial communities, as well as other living organisms, i.e., plants [42]. However, the bioactivity, mobility, and aggregation of AgNPs in the soil depend mostly on the ionic strength of soil but also on the presence of organic matter, humic and fluvic acids, and pH [41]. Most of the studies evaluating the toxicity of AgNPs and nanoparticles in general (including the present study), consider the direct exposure of selected organisms to NPs putting aside non-targeted organisms and characteristics of the environment which can also affect the behavior of nanoparticles [41,45]. Therefore, the pathway of AgNP release, absorption, and fate in the soil/plant systems needs more studies.

Given the promising results regarding the use of nanoparticles in the protection of peas against *D. pinodes* and *F. avencaeum*, it is necessary to further investigate their fungistatic effect and the impact on peas (yield and its potential contamination with nanoparticles) in greenhouse and field experiments, but the impact of different forms of bio-AgNPs application on the environment should also be considered.

### 2.3. Changes in Polar Metabolites Under Fungal Infection

For further analysis of the effect of seed priming on seedlings’ metabolic profiles under fungal infections, plants developed from seeds primed with 100 mg/L of bio-AgNPs were chosen as this was the highest concentration of nanoparticles that did not negatively affect seedling growth and development and effectively reduced infections of both *D. pinodes* and *F. avenaceum*. Infection of 7-day-old pea seedlings by both pathogens negatively affected the seedling growth during the next 14 days. The length of shoot and FW of shoot and roots of infected seedlings developing from water-primed seeds were significantly (*p* ≤ 0.05) lower than in uninfected seedlings (Table S3).

In the case of bio-AgNP priming, fungal infection of seedlings caused only a reduction in the length of the primary root. Other parameters were not affected by infection with either *D. pinodes* or *F. avenaceum* compared to uninfected seedlings (Table S3). Moreover, the application of bio-AgNPs for seed priming contributed to the alleviation of negative effects on shoot’s length and FW, and DW of roots of seedlings infected with *D. pinodes*. In the case of *F. avenaceum* infection, nano-priming alleviated the reduction of shoot length, FW of seedlings, and DW of shoot and cotyledon compared to infected seedlings developed from water-primed seeds (Table S3). Therefore, nano-priming with bio-AgNPs at a concentration of 100 mg/L did not negatively affect seedling growth and development. Moreover, this seed treatment reduced the negative effects of seedling infection by *D. pinodes* and *F. avenaceum*, similar to foliar treatment with bio-AgNPs at concentrations of 100 and 200 mg/L [23].

#### 2.3.1. Effects of Nano-Priming and Fungal Infection on Seedling Metabolic Profile

In the tissues of 21-day-old uninfected pea seedlings developed from water-primed seeds (control), 39 metabolites were identified. They were soluble carbohydrates (fructose, galactitol, glucose, maltose, mannitol, *myo*-inositol, raffinose, stachyose, sucrose, trehalose, verbascose, erythronic acid, and gluconic acid), amino acids (alanine, asparagine, aspartic acid, β-alanine, γ-aminobutyric acid (GABA), glutamic acid, homoserine, hydroxyproline, isoleucine, lysine, phenylalanine, proline, serine, threonine, tyrosine, tryptophan, and valine), organic acids (butyric acid, citric acid, lactic acid, malic acid, malonic acid, oxalic acid, propionic acid, and succinic acid), and the remaining compounds (phosphoric acid and urea; Tables S4–S9).

The metabolic profiles of 21-day-old control seedlings are consistent with our previous reports of metabolic profiles of 5–7-day-old pea seedlings [46,47,48], 22-day-old pea seedlings [23] as well as shoots of mature pea plants [49,50]. Moreover, the trace amounts of raffinose family oligosaccharides (RFOs), such as raffinose, stachyose (in seedlings and cotyledons), and verbascose (only in cotyledons), and small amounts of sugar acids, such as galactitol and mannitol, were detected (Tables S4–S9). The mentioned compounds (RFOs) were not observed in the metabolic profile of 22-day-old pea seedlings, in our recent publication [23], presumably due to their low content. Additionally, it should be noted that in all tissues of seedlings, an unknown soluble carbohydrate (peak no. 4 in Figure S4) was detected. However, the authors were unable to identify this compound using available analytical standards as well as the gas chromatography coupled with mass spectrometry (GC-MS) method (not presented data). Thus, only the average content of this compound (estimated based on the conversion factor for the compound with the closest retention time) was listed in the tables in the Supplementary Materials file (Tables S4–S9), but it was not included in the pool of total identified polar metabolites. The isolation, purification, and assessment of the chemical structure of this compound could be important for the characterization of the pea metabolome.

**Figure 4:**
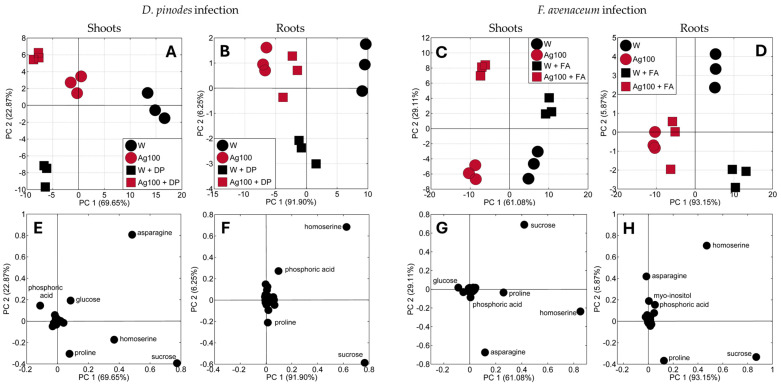
PCA score (**A**–**D**) and loading plots (**E**–**H**) of the metabolic profiles of the shoots and roots of 21-day-old seedlings of pea (*Pisum sativum* L.) developed from seeds after water and bio-AgNPs priming, 14 days after *D. pinodes* (**A**,**B**,**E**,**F**) or *F. avenaceum* (**C**,**D**,**G**,**H**) inoculation. Abbreviations: W and W + DP/FA—uninfected seedlings and seedlings infected with *D. pinodes*/*F. avenaceum*, respectively, grown from water-primed seeds; Ag100 and Ag100 + DP/FA—uninfected seedlings and seedlings infected with *D. pinodes*/*F. avenaceum*, respectively, grown from seeds primed with bio-AgNPs at a concentration of 100 mg/L.

The principal component analysis (PCA) of all identified polar metabolites of the shoots and roots of seedlings grown from water- or nano-primed seeds, both infected and uninfected, clearly showed differences in the data distribution (Figure 4). The shifts in the samples’ distributions were related to the type of seed priming, the seedling’s tissue, and fungal infection. The samples of shoots and roots of seedlings developed from water-primed seeds were separated from those developed from bio-AgNPs-primed seeds, regardless of fungal infection (Figure 4A–D).

Moreover, the fungal infection by both pathogens caused a separation of the samples from uninfected seedlings. According to the PCA loading plots, the distributions of the shoots and roots samples were mostly related to the differences in the concentrations of asparagine, sucrose, homoserine, proline, and phosphoric acid (Figure 4E–H).

The distribution of cotyledon samples was similar in the case of both pathogens’ infections. Samples of seedlings from water- and nano-primed seeds were separated from each other according to PC2 (sharing 6.83 and 6.88% of variance for *D. pinodes* and *F. avenaceum* infection, respectively). However, the separation of cotyledon samples of seedlings (from bio-AgNPs-primed seeds) infected and uninfected is clearer in the case of *D. pinodes* infection (PC1 sharing 90.40%; Figure S5A) than for *F. avenaceum* infection (PC1 90.01%; Figure S5B). The sample distribution was influenced by the differences in the concentrations of asparagine, GABA, homoserine, fructose, and sucrose (Figure S5C,D).

A similar set of differentiating metabolites, mainly asparagine, homoserine, and sucrose, was also observed in our previous study [23]. Moreover, in the present study, glucose was a differentiating metabolite only in roots in the case of infection of both pathogens, as in a recent study. The major difference is that proline and phosphoric acid are differentiators in shoots and roots of seedlings developed from primed seeds after infection of both *D. pinodes* and *F. avenaceum*, whereas in the foliar treatment experiment, proline distinguished only samples of seedlings infected with *F. avenaceum* and phosphoric acid only samples infected with *D. pinodes* [23]. In cotyledons of seedlings after seed priming one of the differentiating metabolites was GABA instead of glucose as in the case of seedlings after foliar treatment from the previous study [23].

#### 2.3.2. Changes in the Concentrations of Polar Metabolites

In 21-day-old control seedlings, the concentration of total identified polar metabolites (TIPMs) was higher in cotyledons (175.42 mg/g DW) than in shoots (157.49 mg/g DW) or roots (87.83 mg/g DW, Table 2).

Amino acids were a major fraction of metabolites in shoots and roots, sharing 59 and 53% of TIPMs, respectively, whereas in cotyledons it stands for 22% of TIPMs (Tables S4–S9). Soluble carbohydrates dominated in cotyledons (71% of TIPMs) but in shoots and roots they were the second most abundant fraction (33% of TIPMs; Tables S4–S9). Regardless of the analyzed pea seedling tissues, among carbohydrates quantitatively dominated sucrose, the major amino acids were asparagine and homoserine, whereas phosphoric acid dominated among the remaining compounds (Tables S4–S9). Citrate, malate, and oxalate were the dominant organic acids in shoots, roots, and cotyledons, respectively (Tables S4–S9). Some metabolites, such as erythronic acid, lysine, and tryptophan were found in cotyledons only (Tables S4–S9). The seed nano-priming and seedling infection with *D. pinodes* or *F. avenaceum* considerably affected the concentration of TIPMs in pea shoots and roots, and also in cotyledons, but to a lesser extent (Table 2).

**Figure 5:**
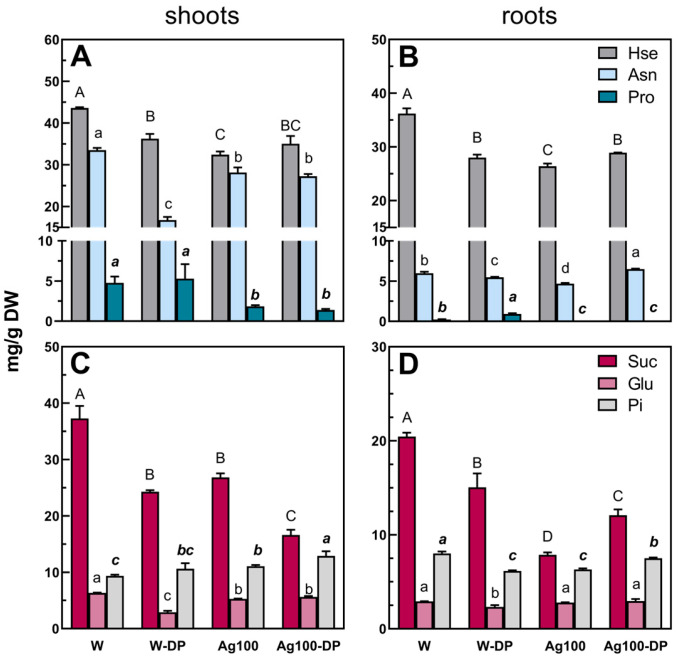
The concentrations of selected metabolites: homoserine (Hse), asparagine (Asn), proline (Pro), sucrose (Suc), glucose (Glu), and phosphoric acid (Pi) in the shoots (**A**,**C**) and roots (**B**,**D**) of pea (*Pisum sativum* L.) developed for 21 days (from water- or bio-AgNPs-primed seeds) without infection or for 14 days after inoculation of 7-day-old seedlings with *D. pinodes*. Values (in mg/g DW) are the means of 3 replicates + SD. The same letters (A–D; a–d; ***a–c***; separately for each metabolite) above the bars indicate statistically insignificant (*p* ≤ 0.05) differences based on two-way ANOVA and Tukey’s post–hoc test. Abbreviations: W and W-DP—non-infected seedlings and seedlings infected with *D. pinodes*, respectively, grown from water-primed seeds; Ag100 and Ag100-DP—non-infected seedlings and seedlings infected with *D. pinodes*, respectively, grown from seeds primed with bio-AgNPs at a concentration of 100 mg/L.

**Figure 6:**
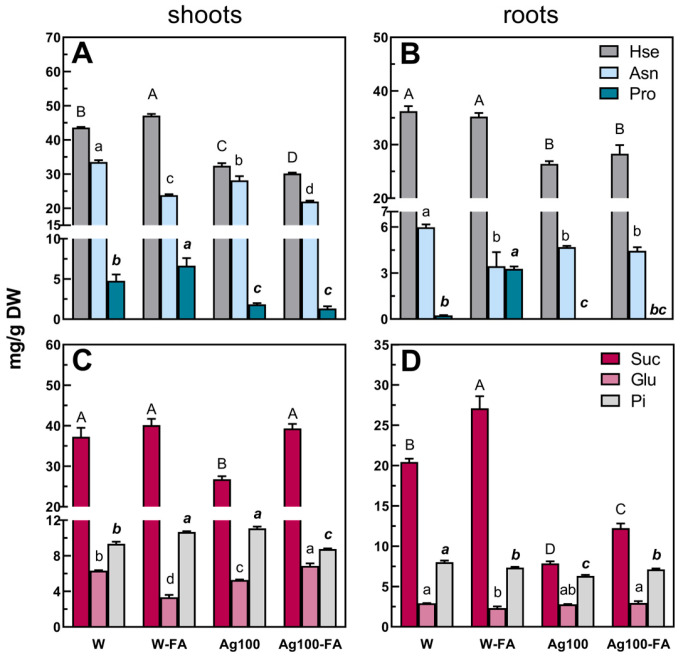
The concentrations of selected metabolites: homoserine (Hse), asparagine (Asn), proline (Pro), sucrose (Suc), glucose (Glu) and phosphoric acid (Pi) in the shoots (**A**,**C**) and roots (**B**,**D**) of pea (*Pisum sativum* L.) developed for 21 days (from water- or bio-AgNPs-primed seeds) without infection or for 14 days after inoculation of 7-day-old seedlings with *F. avenaceum*. Values (in mg/g DW) are the means of 3 replicates + SD. The same letters (A–D; a–d; ***a–c***; separately for each metabolite) above the bars indicate statistically insignificant (*p* ≤ 0.05) differences based on two-way ANOVA and Tukey’s post–hoc test. Abbreviations: W and W-FA—non-infected seedlings and seedlings infected with *F. avenaceum*, respectively, grown from water-primed seeds; Ag100 and Ag100-FA—non-infected seedlings and seedlings infected with *F. avenaceum*, respectively, grown from seeds primed with bio-AgNPs at a concentration of 100 mg/L.

The above-mentioned differences in the TIPMs (and their fractions) resulted mainly from changes in the content of some amino acids (homoserine, asparagine, and proline), sugars (sucrose, glucose), and phosphoric acid (Figure 5 and Figure 6), identified as the major compounds differentiating samples according to PCA loading plots (Figure 4E–H).

Homoserine (Hse) and asparagine (Asn) were the most abundant amino acids in pea seedling tissues. The concentration of Asn decreased in pea seedlings developed from water-primed seeds after an infection caused by both *D. pinodes* and *F. avenaceum* (Figure 5A,B and Figure 6A,B; Tables S4, S5, S7 and S8). A similar response was observed in Hse in seedlings infected with *D. pinodes* (Figure 5A,B; Tables S4 and S5) but not in the case of *F. avenaceum* infection. Homoserine concentration in shoots increased (Figure 6A; Table S7), whereas in roots, it remained at the same level as in uninfected seedlings grown from water-primed seeds (Figure 6B; Table S8). Previous findings also showed that in pea seedlings 14 days after inoculation with *D. pinodes* Hse content decreased, whereas *F. avenaceum* infection did not affect its concentration in roots. Similarly, Asn depletion in shoots 14 days after pea seedlings inoculation with both of the mentioned pathogens was also observed [23]. In seedlings developed from nano-primed seeds, concentrations of Hse and Asn after *D. pinodes* infection increased (in roots) or remained at a similar level (in shoots) compared to uninfected seedlings from nano-primed seeds (Figure 5A,B; Tables S4 and S5). Whereas in the case of the infection with the second pathogen, Hse and Asn concentrations decreased in shoots (Figure 6A; Table S7) but no changes were observed in roots (Figure 6B; Table S8).

The high content of Asn and Hse is related to their important functions in plants. The main role of Hse is the mobilization and transport of carbon and nitrogen reserves [51]. Asn is an amino acid whose main role is the storage and transport of nitrogen in plants’ tissues [52,53]. Asn and Hse are also considered as host signals that induce *in planta* expression of pectate lyase coded by *pel*D of *Nectria haematococca* (current name: *Fusarium haematococcum*) during pea infection [54]. Moreover, Asn is involved in the synthesis of other amino acids, such as Hse, which is an intermediate in the biosynthesis of methionine and isoleucine [52,53] that can be further utilized for secondary metabolites synthesis [55,56] and thus counteract the pathogens’ attack [57,58].

Proline (Pro) content increased after infection with *D. pinodes* (in roots; Figure 5B) and *F. avenaceum* (both in shoots and roots; Figure 6A,B), but only in seedlings developed from water-primed seeds. Accumulation of this amino acid in roots corresponds with our previous findings [23]. However, no Pro accumulation was observed in shoots after by *D. pinodes* infection in the present as well as in our prior study [23]. This suggests that Pro could be accumulated in the aboveground parts of pea plants as a first step of response to this pathogen, up to 36 h post-inoculation as reported by Desaleng et al. [59] and Turetschek et al. [60] but not necessarily after a longer time of infection.

In the case of seedlings developed from bio-AgNPs-primed seeds, the Pro level did not change after infection. Moreover, it was significantly lower than that found in seedlings developed from water-primed seeds, both infected and uninfected (Figure 5A,B). Reduced Pro concentration was also observed in seedlings infected with *D. pinodes* and *F. avenaceum* after short-term immersion in bio-AgNPs compared to control-infected seedlings, but the content of this amino acid was at a similar level as in control uninfected seedlings [23]. Proline is a proteinogenic stress-related amino acid. Its content increases in plants’ tissues due to abiotic stresses (such as drought or salinity stress) but also due to biotic stresses. Pro can act as an osmolyte, chemical chaperon, ROS scavenger, or metal chelator. However, its metabolism is also involved in energy production, redox regulation, and ROS signaling [61]. The lower content of Pro in seedlings grown from nano-primed seeds (infected and uninfected) in comparison to control seedlings indicates that bio-AgNPs-priming triggered processes of Pro utilization rather than synthesis or that Pro synthesis was impaired. Thus, the lower content of Pro might be caused by its catabolism to provide more energy for seedlings via the respiratory electron transport chain [62,63]. Moreover, Pro oxidation causes ROS production (through NADPH oxidase) which can promote a hypersensitive response or programmed cell death by salicylic acid (SA) pathway stimulation [62].

Glucose content decreased in roots and shoots developed from seeds after water priming after both *D. pinodes* and *F. avenaceum* infection and remained at the same level in the case of nano-priming, except for shoots infected with *F. avenaceum*, where glucose content increased in comparison to the nano-primed uninfected control (Figure 5, Tables S2, S3, S5 and S6). Sucrose content decreased in shoots, regardless of the type of seed priming (and roots of seedlings developed from water-primed seeds) after *D. pinodes* infection, whereas in the case of bio-AgNPs-priming—it increased (Figure 5C,D, Tables S2 and S3). In contrast, after *F. avenaceum* infection, its concentration increased in roots, regardless of the type of priming, and in shoots after water priming. In the case of nano-priming, no significant changes in sucrose concentration were observed in shoots (Figure 6C,D, Tables S5 and S6). Moreover, trehalose increased after infection in shoots and roots, but only in infected seedlings developed from water-primed seeds (*D. pinodes* infection—almost 2-fold increase; *F. avenaceum*—2.5-fold increase). No such changes were observed for nano-priming (Tables S2, S3, S5 and S6). Depletion of sucrose in shoots under *D. pinodes* infection was also observed in other studies [23,59,60]. Carbohydrates, mainly sucrose and monosaccharides, act in various ways during infection. Apart from being utilized by phytopathogen, the host’s sugar metabolism is intensively reprogrammed to provide a sufficient C source, energetic substrates, and reducing agents required in the plant’s defense response [58,64,65]. Moreover, sucrose, glucose, and trehalose can act as signaling molecules that induce isoflavonoid synthesis, the expression of pathogen-related genes, phenylalanine lyase, and peroxidase activity, thus promoting secondary metabolites production [64,66].

Phosphoric acid (Pi) was also identified as the samples’ differentiating metabolite (Figure 4E,H). In infected seedlings, developed from water-primed seeds, phosphate content decreased in roots, while increased in shoots (under both infections). Pi concentration increased in seedlings developed from bio-AgNPs primed seeds after infections in comparison to uninfected ones, except shoots after *F. avenaceum* infection (Figure 5C,D and Figure 6C,D). Phosphate homeostasis controlled by phosphate starvation response (PSRs) might be involved in plant immunity. Pi affects the biosynthesis of salicylic acid (SA) and jasmonic acid (JA) which are essential in plant immunity responses and trigger the synthesis of secondary metabolites related to plant immunity [67,68]. Recent studies reported that high phosphate content in *Arabidopsis thaliana* reduces susceptibility to fungal pathogens infection [69] and Pi deficiency increases plant susceptibility [70]. In contrast, it was also shown that phosphate excess in rice can make it more sensitive to pathogen infections [71]. Thus, phosphate can modulate plants’ immunity response, but host–pathogen interactions seem to be an important factor affecting the direction of this response.

Infection of *D. pinodes* did not affect the TIPMs in cotyledons of seedlings developed from water-primed seeds, while *F. avenaceum* infection caused an increase in polar metabolite content, especially TSCs subfraction (Table 2). TIPM content in cotyledons of seedlings developed from bio-AgNPs-primed seeds was similar to those water-primed but TSC concentration was increased, whereas TAAs decreased (Table 2). Pathogen infections negatively affected TIPM content by decreasing soluble carbohydrates and amino acid concentrations (Table 2). Compounds such as homoserine, GABA, asparagine, proline, sucrose, and fructose were identified as the major compounds differentiating samples according to the PCA loading plots (Figure S5C,D).

Regardless of the type of priming, homoserine content decreased, whereas fructose increased after infection caused by each of the pathogens (Figure S6A,B, Tables S6 and S9). The main differences between uninfected cotyledons of seedlings developed from water- and nano-primed seeds were the concentrations of proline, fructose, and galactinol which were lower over 4-, 3-, and 8-fold, respectively, in the case of bio-AgNPs priming (Figure S6, Tables S6 and S9). Proline content in cotyledons changed in the same way as in shoots and roots—in the case of water-priming, its content increased after infections but remained at the same level in cotyledons of seedlings developed from bio-AgNPs-primed seeds. GABA content increased after *D. pinodes* infection regardless of the type of priming in comparison to the respective uninfected controls, whereas after *F. avenaceum* infection some differences were noticed. Asn content decreased after infections, except in cotyledons of seedlings developed from nano-primed seeds infected with *D. pinodes*, where it remained at the same level as in uninfected ones (Figure S6, Tables S6 and S9). Sucrose content decreased after both *D. pinodes* and *F. avenaceum* infection in cotyledons of seedlings developed from nano-primed seeds, whereas no changes (*D. pinodes* infection) or accumulation (*F. avenaceum* infection) were observed in the case of water-priming (Figure S6A,B, Tables S6 and S9).

Enhanced mobilization of storage sources of C and N is needed for the plants to effectively fight the pathogen attack. In addition to their energy and building functions, sugars and amino acids are involved in the regulation of defense signals by influencing phytohormone signaling pathways, producing ROS, or enhancing the expression of defense-related proteins, which may further enhance the production of plant secondary metabolites [58]. Moreover, nano-priming has been shown to enhance plant defense responses by modulating signaling pathways and stimulating and regulating plant secondary metabolism [3,72]. It should also be noted that the metabolic changes under pathogen attack are often organ- and species-specific [59,60,73,74,75].

Infection with *D. pinodes* and *F. avenaceum* affected the metabolic profiles of pea seedlings, forcing them to shift their metabolism to counteract the pathogens’ attack. Seed nano-priming on its own also affected the metabolism of seedlings that developed from such treated seeds. The observed changes lead us to suggest that the response of pea seedlings to both the nanoparticles themselves and the pathogen attack should be seen primarily in changes in secondary metabolism.

## 3. Materials and Methods

### 3.1. Bio-Synthesized Silver Nanoparticles

The silver nanoparticles used in this study are nanocomposites synthesized using a *Lactobacillus paracasei* post-culture medium and were synthesized and fully described by Railean-Plugaru et al. [36]. Nanoparticles’ characteristics like the elemental composition of the bio-synthesized silver nanoparticles, the core size and morphology, the surface composition, interactions between the metallic surfaces and organic ligands, and the content of elemental silver were described previously [23,36]. Briefly, bio-AgNPs are spherical and homogeneous with an average size of 18.25 ± 0.58 nm. The elemental silver content is 9.03 ± 0.02 mg/mL with the total silver content in the nanoparticles 11.25 ± 0.02% by atomic percentage, containing predominantly metallic silver (98.9% of total Ag) rather than the ionic form (1.1%). The organic surface coating of the nanoparticles contains carbon, oxygen, phosphorus, and sulfur. The zeta potential values range from −23 to −41 mV, hydrodynamic diameter of 100 to 150 nm, and a polydispersity index below 0.7.

### 3.2. Preliminary Studies for Priming Conditions

In the first preliminary study, we tested the optimal time of priming for the pea (*Pisum sativum* L.) seed cultivar Nemo, purchased from Danko Hodowla Roślin (Choryń, Poland). Seeds (100 per time point) were covered with 50 mL of double-distilled water and incubated for 0.5, 1, 2, and 4 h at room temperature. After each time point the seeds were dried with paper towels to remove excess water. Then, they were placed on glass petri dishes and dried at 30 °C for 24 h (FD 115, Binder, Tuttlingen, Germany). Dried seeds were germinated for 4 days in rolls of wet filter paper placed in 250 mL glass cylinders (22 °C, in the dark, in a climatic chamber ILW 115-T STD, Pol-Eko-Aparatura, Wodzisław Śląski, Poland); 3 replications of 30 seeds for each time point. Then, the seedlings’ roots and shoot length were measured.

In the second preliminary study, we tested the optimal concentration and time of pea seed priming with bio-AgNPs. The nanoparticles were suspended in double-distilled water via sonication (Sonic-3, 310 W, 40 KHz, POLSONIC, Pałczyński, Poland) for proper nanoparticle distribution (2 times for 30 min) and then used for priming. Seeds (100 seeds per each tested variant) were covered with 50 mL of double-distilled water or bio-AgNPs suspensions at concentrations of 50, 100, and 200 mg/L and incubated for 0.5, 1, and 1.5 h at room temperature. Then, the seeds were dried with paper towels to remove excess water/nanoparticle suspensions and dried at 30 °C for 24 h (FD 115, Binder, Tuttlingen, Germany). Dried seeds were germinated for 4 days as described above in 3 replications of 20 seeds for each variant. Then, the seedlings’ length, fresh and dry weight of roots, epicotyls, and cotyledons were measured.

### 3.3. Plant Material

In the present study, the pea (*Pisum sativum* L.) seed cultivar Nemo, purchased from Danko Hodowla Roślin (Choryń, Poland) was used. Seeds (200 per variant) were primed with double-distilled water and bio-AgNPs suspensions at concentrations of 50 and 100 mg/L for 1h (conditions as in preliminary studies). Additionally, commercial seed coat (CSC) Maxim 025 FS with fludioxonil 25 g/L (2.38%) (Syngenta, Warsaw, Poland) was used as a control of the effectiveness of nanoparticles in pea protection against selected pathogens. The CSC was used in accordance with the manufacturer’s recommendations. After priming, dry seeds were germinated for 4 days in rolls of wet filter paper placed in 250 mL glass cylinders (22 °C, in the dark, in a climatic chamber Snijders Scientific, Tilburg, The Netherlands). Then, they were transferred to 10 mL probes with distilled water with the roots immersed in water and incubated at 22 °C (day/night, 12 h/12 h; climatic chamber Snijders Scientific, Tilburg, The Netherlands) for the next three days. Water in the probes with seedlings was replenished daily. The 7-day-old healthy seedlings with properly developed epicotyl and primary roots were used for the infection experiments.

### 3.4. Fungal Material

*Didymella pinodes* strain no. CBS 107.45, purchased from the Westerdijk Fungal Biodiversity Institute (Utrecht, The Netherlands), and *Fusarium avenaceum* strain no. A232/2019, acquired from the collection of the Department of Entomology, Phytopathology, and Molecular Diagnostics, the University of Warmia and Mazury (Olsztyn, Poland) were used in this study. Cultures of *D. pinodes* and *F. avenaceum* were carried out in petri dishes on a potato-dextrose agar (PDA; potato extract 20%, glucose 1.6%, agar 1.8%) at 22 °C (day/night 12 h/12 h, in a climatic chamber). The preparation of fungal spores for plant infection was performed as described previously [23]. The spore suspensions were diluted to 1 × 10^7^ CFU/mL (measured using a Brücker hemocytometer, Heinz Herenz Medizinalbedarf GmbH, Hamburg, Germany) and used for infection of 7-day-old pea seedlings. Tween80 was added to the spore suspension to improve adhesion at a final concentration of 0.1% in the suspension.

### 3.5. Plant Infection

The 7-day-old healthy seedlings, grown from primed seeds (3 replications of 10 seedlings for each priming variant), with properly developed epicotyl and primary roots were used for the infection. Pea seedlings were inoculated with spores of *D. pinodes* (base of shoot and shoot) or *F. avenaceum* (base of the shoot and root) prepared as described in Section 3.4. The seedlings were incubated in a climatic chamber for 14 days (temp. 22 °C, day/night 12 h/12 h), and water was replenished daily. After 14 days the morphology of the plants (FW, DW, and the length of shoots and primary roots) was measured, and the disease index was determined. Seedlings were frozen in liquid nitrogen and stored at −80 °C. Half of the pea seedlings from each replication were used for qPCR analysis (whole seedlings) and the other half for the metabolomic analysis (divided into shoots, roots, and cotyledons).

### 3.6. Disease Index

The assessment of the health of the pea seedlings was performed as described previously [23], performed according to the modified scale of Hillstrand and Auld [76], and the disease index of the pea was calculated according to the McKinney Formula (2) [77]:

DI (%) = (Σ (a × b) × 100%)/(N × I) (1)

DI—the disease index; Σ (a × b)—the sum of the products after multiplying the number of plant organs examined by the given degree of infestation; N—the total number of organs examined; I—the highest degree of infestation on the scale.

### 3.7. qPCR Analysis

The collected tissues (whole seedlings) were homogenized in liquid nitrogen using a mortar and pestle. The isolation of gDNA was performed using gDNA-isolation for Maxwell^®^ 16 FFS (Promega, Madison, WI, USA) with minor modifications [23]. Matrices with high-quality parameters, measured using a NanoDrop ND 2000c spectrophotometer (Thermo Scientific, Waltham, MA, USA), were used in further studies. The qPCR was performed in triplicate for each test variant. The qPCR was performed using primers specific for *D. pinodes* [78] and *F. aveneceum* [79] (Table 3), using a 7500 Fast Real-Time PCR System (Applied Biosystems, ThermoFisher Scientific, Waltham, MA, USA). The reaction mixture contained 12.5 µL of TaqMan Fast Universal PCR Master Mix (Applied Biosystem, ThermoFisher Scientific, Waltham, MA, USA), 10 pM probes labeled at the ends with 5′-FAM and 3′-MGB as a quencher, 10 pM primers, 4.5 µL of deionized water, 5 µL of gDNA, and a total volume of 25 μL. The amplification conditions were the same as those described by Okorski et al. [80]. The negative control was deionized, sterile water, while the positive control was gDNA extracted from *D. pinodes* and *F. avenaceum* 21-day-old cultures. Quantitative calculations of qPCR were performed according to the method described by Livak and Schmittgen [81].

### 3.8. Polar Metabolite Analyses

Seedlings after nano-priming that showed the highest effectiveness in protecting against *D. pinodes* and *F. avenaceum* were selected for metabolome analyses.

Tissue samples of infected seedlings were lyophilized and pulverized in a mixer mill (MM200, Retsch, Haan, Germany). Extraction and separation of polar metabolites were performed according to Szablińska-Piernik and Lahuta [49]. Briefly, 40–42 mg of milled tissue (at least 3 biological replicates) was extracted with 900 μL of 50% methanol at 70 °C for 30 min with continuous shaking (500 rpm; Thermo-shaker MS-100 ALLSHENG, Hangzhou, China). Ribitol was used as the internal standard (100 μL of 1 mg/mL ribitol added to the extraction mixture). Then, the homogenates were cooled on ice and centrifuged (20,000× *g* at 4 °C for 20 min). The supernatants were mixed with cold chloroform to remove non-polar compounds. The samples were then dried in chromatographic vials and stored in desiccators until chromatographic analysis. Seedlings metabolic profiling was performed using a gas chromatograph GC2010 Nexia (Shimadzu, Kyoto, Japan) with a flame ionization detector (FID) for robust quantitative analyses of metabolites and a gas chromatograph coupled with mass spectrometry (QP-GC-2010, Shimadzu, Japan) to confirm accurate metabolite identification. After two-step derivatization (with O-methoxamine hydrochloride and a mixture of N-methyl-N-trimethylsilyl-trifluoroacetamide (MSTFA) with pyridine (1:1, *v*/*v*)), trimethylsilyl (TMS)-derivatives were separated on a capillary column ZEBRON ZB-5MSi Guardian (length 30 m, diameter 0.25 mm, film 0.25 μm; Phenomenex, Torrance, CA, USA). Metabolites were identified and characterized by the comparison of their retention times (RT), retention indices (RI, determined according to the saturated hydrocarbons), and mass spectra of original standards derived from Sigma-Aldrich (Sigma-Aldrich, Merck, Burlington, MA, USA) and the NIST library (National Institute of Standards and Technology, Gaithersburg, MD, USA).

The separation of TMS derivatives of carbohydrates on a 30 m column did not allow precise identification of soluble carbohydrates. Therefore, sugar derivatization and separation was repeated on a 15 m column, according to the method specific for soluble carbohydrates analysis [82].

### 3.9. Soluble Carbohydrates Analyses

Milled tissue (40–42 mg), from the same samples of lyophilized plant material as for previous polar metabolite extraction, was extracted with 800 μL of 50% ethanol with 100 μL of Xylitol (1 mg/mL) as internal standard, at 90 °C for 30 min with continuous shaking (500 rpm; Thermo-shaker MS-100 ALLSHENG, Hangzhou, China). The homogenates were cooled on ice and centrifuged (20,000× *g* at 4 °C for 20 min). The aliquots of supernatants (400 μL) were filtered using micro-spin filters (PVDF, 0.2 μm, Thermo Fisher Scientific, Loughborough, UK). After filtration, 200 μL aliquots of the samples were dried in chromatographic vials and stored in desiccators until chromatographic analysis.

Carbohydrates were derivatized with a mixture of trimethylsilyl imidazole and pyridine (1:1, *v*/*v*). TMS derivatives of soluble carbohydrates were analyzed using a gas chromatograph (GC 2010, Shimadzu, Japan) with FID, separated on a capillary column Zebron ZB-1 (length 15 m, diameter 0.25 mm, film 0.1 μm; Phenomenex, Torrance, CA, USA) as described by Lahuta et al. [70]. Carbohydrates were identified by the comparison of their RT to original standards from Sigma-Aldrich (Sigma-Aldrich, Merck, Burlington, MA, USA) and quantified from the standard curves using original standards purchased from Sigma-Aldrich (Sigma-Aldrich, Merck, Burlington, MA, USA). Data for the concentration of soluble carbohydrates excluding sugar acids (data for erythronic and gluconic acid come from metabolite analyses) were added to the metabolite profiling results.

### 3.10. Statistical Analyses

The results are the mean of 3 independent replicates, and they were subjected to one-way ANOVA with a post-hoc test (Tukey, if overall *p* ≤ 0.05) or two-way ANOVA with a post-hoc test (Tukey, if overall *p* ≤ 0.01 for the disease index and qPCR analyses and *p* ≤ 0.05 for other data), using Statistica software (version 12.0; StatSoft, Tulsa, OK, USA). Graphs were prepared using GraphPad Prism, version 8 (GraphPad Software, San Diego, CA, USA). Multivariate statistics of metabolomic data were analyzed using principal component analysis (PCA) and performed using COVAIN [83], a MATLAB toolbox including a graphical user interface (MATLAB version 2013a; Math Works, Natick, MA, USA).

## 4. Conclusions

Both tested concentrations of bio-AgNPs used for pea seed priming were found to be effective in reducing infections of both *D. pinodes* and *F. avenaceum* and did not negatively affect the growth and development of seedlings. Therefore, bio-AgNPs used for seed priming as well as for foliar treatment have the potential to be successfully used for fungal disease management caused by mentioned phytopathogens. However, the potential risks associated with the application of silver nanomaterials in agriculture [84] and various industries [85] are still a subject of ongoing debate and need active research. Thus, future studies are justified to investigate the effects of bio-AgNPs in the longer term, in greenhouse or field experiments over several seasons. This would allow determining the validity of using silver nanoparticles in plant protection and their impact on the environment.

Pathogens’ infection shifted/rearranged pea seedlings’ metabolic profiles. The major changes were observed in the metabolism of amino acids (asparagine, homoserine, and proline) and carbohydrates (mainly sucrose and glucose). Changes in C and N management suggest increased demand for plants for energy and building substrates. Moreover, the mentioned metabolites may be involved in phytohormone signaling pathways, ROS production, or enhancement of the expression of defense-related proteins. In turn, it could result in changes in the expression and accumulation of secondary metabolites. Thus, results of analyses of primary and secondary metabolism may, in the future, provide a more complete picture of the response of pea seedlings to attack by fungal pathogens. Such data could identify metabolic markers of pea response/or resistance to these pathogens.

## 5. Patents

The details of the application of silver nanoparticles synthesized with lactic acid bacteria as a fungistatic agent for the protection of legumes (*Fabaceae*), especially field pea (*Pisum sativum* L.) against *Didymella pinodes* mentioned in this study is covered by a patent application in Poland no. P.445104.

## Figures and Tables

**Table 1:** Fresh weight (FW), dry weight (DW), and lengths of epicotyl (E), root (R), and 2 cotyledons (C) of 4-day-old pea seedlings developed from seeds primed with water as control and bio-AgNPs at concentrations of 50, 100, and 200 mg/L for 0.5, 1 and 1.5 h. Means of 3 replicates. The same letters (a–e) indicate statistically insignificant (*p* ≤ 0.05) differences (valid separately for data in rows) based on two-way ANOVA and Tukey’s post-hoc test.

		Water Priming			Bio-AgNPs Priming								
					50 mg/L			100 mg/L			200 mg/L		
Time of Priming		0.5 h	1 h	1.5 h	0.5 h	1 h	1.5 h	0.5 h	1 h	1.5 h	0.5 h	1 h	1.5 h
Length (mm)	E	13.0 ^ab^	12.4 ^ab^	15.3 ^a^	11.4 ^abc^	10.1 ^abcd^	6.2 ^cde^	6.8 ^de^	9.5 ^bcde^	8.9 ^bcde^	4.8 ^de^	6.2 ^de^	4.8 ^e^
	R	32.4 ^a^	32.5 ^a^	32.6 ^ab^	30.4 ^abc^	23.8 ^bcd^	23.3 ^cd^	22.2 ^d^	25.9 ^abcd^	24.0 ^bcd^	24.2 ^bcd^	27.1 ^abcd^	22.5 ^d^
FW (mg)	E	38.1 ^ab^	45.6 ^ab^	30.8 ^b^	34.2 ^ab^	40.7 ^ab^	38.0 ^ab^	44.9 ^ab^	54.9 ^a^	38.8 ^ab^	37.6 ^ab^	37.8 ^ab^	34.5 ^ab^
	R	59.9 ^ab^	64.7 ^a^	60.1 ^ab^	62.1 ^a^	45.7 ^c^	47.6 ^bc^	42.7 ^c^	45.7 ^c^	45.3 ^c^	44.4 ^c^	48.1 ^bc^	41.4 ^c^
	C	296.8 ^ab^	331.1 ^a^	278.9 ^b^	291.8 ^ab^	312.9 ^ab^	303.9 ^ab^	316.4 ^ab^	309.9 ^ab^	304.0 ^ab^	306.9 ^ab^	296.4 ^ab^	321.2 ^a^
DW (mg)	E	4.6 ^ab^	4.3 ^ab^	3.0 ^b^	3.3 ^ab^	4.6 ^ab^	3.9 ^ab^	4.4 ^ab^	5.1 ^a^	3.9 ^ab^	3.7 ^ab^	3.8 ^ab^	3.5 ^ab^
	R	5.3 ^a^	5.4 ^a^	5.4 ^a^	4.7 ^ab^	3.7 ^c^	4.0 ^bc^	3.5 ^c^	3.7 ^c^	3.6 ^c^	3.6 ^c^	3.9 ^bc^	3.4 ^c^
	C	121.4 ^a^	122.3 ^a^	111.3 ^a^	112.7 ^a^	122.2 ^a^	116.2 ^a^	121.9 ^a^	117.5 ^a^	118.1 ^a^	118.1 ^a^	117.1 ^a^	126.5 ^a^

**Table 2:** The concentration of total identified polar metabolites (TIPMs), including total soluble carbohydrates (TSCs), total amino acids (TAAs), total organic acids (TOAs), and total remaining compounds (TRCs) in the shoots, roots, and cotyledons of pea seedlings (*Pisum sativum* L.), developing (from water- and bio-AgNPs-primed seeds) for 21 days without infection (uninfected) or for 14 days after inoculation of 7-day-old seedlings with *D. pinodes* or *F. avenaceum* (infected). Means of 3 replicates. The same letters by the values indicate statistically insignificant differences (*p* ≤ 0.05) based on two-way ANOVA analysis and Tukey’s post-hoc test (valid in rows).

		Seedlings Developed from Seeds					
		Water-Primed			Bio-AgNPs-Primed		
		Uninfected	Infected with		Uninfected	Infected with	
			*D. pinodes*	*F. avenaceum*		*D. pinodes*	*F. avenaceum*
shoots	TIPMs	157.49 ^a^	121.96 ^c^	158.11 ^a^	128.93 ^bc^	123.73 ^bc^	129.69 ^b^
	TSCs	51.49 ^a^	34.53 ^c^	50.76 ^a^	39.34 ^b^	29.39 ^d^	54.10 ^a^
	TAAs	92.35 ^a^	69.98 ^b^	88.97 ^a^	73.38 ^b^	75.35 ^b^	62.36 ^c^
	TOAs	4.06 ^c^	5.50 ^b^	6.45 ^a^	3.39 ^d^	4.09 ^c^	2.87 ^d^
	TRCs	9.59 ^cd^	10.80 ^bc^	10.84 ^bc^	11.52 ^b^	13.45 ^a^	9.04 ^d^
roots	TIPMs	87.83 ^a^	68.76 ^b^	90.35 ^a^	57.75 ^c^	70.17 ^b^	66.88 ^b^
	TSCs	29.04 ^b^	21.50 ^c^	33.48 ^a^	14.48 ^d^	19.85 ^c^	20.01 ^c^
	TAAs	46.23 ^a^	37.37 ^bc^	44.96 ^a^	34.28 ^c^	40.00 ^b^	37.12 ^bc^
	TOAs	4.45 ^a^	3.67 ^b^	4.43 ^a^	2.46 ^c^	2.58 ^c^	2.49 ^c^
	TRCs	8.11 ^a^	6.22 ^d^	7.48 ^bc^	6.53 ^d^	7.75 ^b^	7.26 ^c^
cotyledons	TIPMs	175.42 ^bc^	177.59 ^bc^	194.29 ^a^	181.68 ^b^	154.68 ^d^	169.16 ^c^
	TSCs	125.25 ^c^	127.10 ^c^	150.72 ^a^	142.50 ^b^	118.98 ^c^	137.50 ^b^
	TAAs	38.09 ^a^	38.03 ^a^	34.04 ^ab^	29.10 ^bc^	26.30 ^cd^	22.57 ^d^
	TOAs	4.89 ^b^	6.19 ^a^	3.67 ^c^	3.68 ^c^	3.86 ^c^	3.59 ^c^
	TRCs	7.19 ^a^	6.27 ^ab^	5.86 ^b^	6.39 ^ab^	5.54 ^b^	5.47 ^b^

**Table 3:** qPCR primers and probes used for the identification of *F. avenaceum* and *D. pinodes*.

Genotype/Gene	Primer/Probe	Sequence (5′-3′)	Regression Equation, Efficiency of qPCR (E)	Reference
*D. pinodes* ITS	Forward	5′-AGAGACCGATAGCGCACAAG-3′	y = −3.77x + 23.9 R^2^ = 0.96; E = 91.9	[78]
	Reverse	5′-AGTCCAGGCTGGTTGCAGGA-3′		
	Probe	FAM—CATGTACCTCTCTTCGGG—MGB		
*F. avenaceum Esyn1*	Forward	5′-AGCAGTCGAGTTCGTCAACAGA-3′	y = −3.44x + 19.7 R^2^ = 0.99; E = 95.3	[79]
	Reverse	5′-GGCYTTTCCTGCGAACTTG-3′		
	Probe	FAM—CCGTCGAGTCCTCT—MGB		

## Data Availability

The data presented in this study are available in this article and in the Supplementary Materials.

## Supplementary Materials

The following supporting information can be downloaded at: https://www.mdpi.com/article/10.3390/ijms252111402/s1.
