# Supplementary material for: Seeds Priming with Bio-Silver Nanoparticles Protects Pea (Pisum sativum L.) Seedlings Against Selected Fungal Pathogens
Source: Int J Mol Sci. 2024 Oct 23;25(21):11402. doi: 10.3390/ijms252111402 (PMC11546818; doi:10.3390/ijms252111402)
Supplement: Supplementary file 1 [file ijms-25-11402-s001.zip › ijms-3249054-supplementary.pdf]

Supplementary materials

Tables

Table S1. Epicotyl and root lengths of 4-day-old pea seedlings developed from seeds primed with water for 0.5, 1, 2, or 4 hours. Means of 3 replicates. The same letters (a–c) indicate statistically insignificant (p < 0.05) differences (valid separately for roots and shoots) based on one-way ANOVA and Tukey's post-hoc test.

		Time of pea seeds water priming			
		0.5 h	1 h	2 h	4 h
Length (mm)	Epicotyl	23.2^{a}	17.5^{ab}	17.6^{ab}	11.1^{b}
	Root	68.2^{a}	58.8^{ab}	48.4^{bc}	33.5^{c}

Table S2. Fresh weight (FW), dry weight (DW), and lengths of epicotyl and root lengths of 21-day-old pea seedlings developed from seeds primed with water or bio-AgNPs at concentrations of 50 mg/L and 100 mg/L for 1 hour. Means of 3 replicates. The same letters (a–c) indicate statistically insignificant (p < 0.05) differences (valid in rows) based on one-way ANOVA and Tukey's post-hoc test.

		water priming	bio-AgNPs priming	
			50 mg/L	100 mg/L
Length (mm)	Shoot	48.2^{a}	51.1^{a}	52.2^{a}
	Root*	70.6^{b}	66.7^{b}	88.9^{a}
FW (mg)	Shoot	288.7^{a}	262.1^{a}	342.0^{a}
	Root	264.8^{a}	253.3^{a}	263.8^{a}
	Cotyledon	251.9^{b}	314.9^{a}	264.1^{b}
DW (mg)	Shoot	48.1^{a}	51.3^{a}	47.9^{a}
	Root	20.8^{a}	19.0^{a}	22.6^{a}
	Cotyledon	76.7^{a}	76.5^{a}	68.0^{a}

*- the length of the primary root

Table S3. The length of shoot and primary root, and the fresh weight (FW) and dry weight (DW) of shoot, roots, and cotyledons of pea seedlings (from seeds primed with water or bio-AgNPs at 100 mg/L for 1 h), developed for 14 days without fungal infection or inoculated with *D. pinodes* or *F. aveanceum* at 7th day after germination. Means of 3 replicates. The same letters (a–c) indicate statistically insignificant (p < 0.05) differences (valid separately for data in rows) based on two-way ANOVA and Tukey's post-hoc test.

			Water priming		bio-AgNPs priming 100 mg/L for 1h	
			No infection	Infection	No infection	Infection
D. pinodes	Length (mm)	Shoot	48.2^{ab}	41.6^{b}	52.2^{ab}	52.1^{a}
		Root*	70.6^{c}	79.7^{b}	88.9^{a}	74.4^{bc}
	FW (mg)	Shoot	288.7^{a}	155.8^{b}	342.0^{a}	288.0^{a}
		Roots	264.8^{a}	219.0^{b}	263.6^{a}	251.9^{ab}
		Cotyledons	503.8^{a}	481.4^{a}	528.2^{a}	517.6^{a}
	DW (mg)	Shoot	48.1^{a}	44.3^{a}	47.9^{a}	53.9^{a}
		Roots	20.8^{a}	15.5^{b}	22.6^{a}	21.3^{a}
		Cotyledons	153.8^{a}	158.0^{a}	136.0^{a}	137.4^{a}
F. avenaceum	Length (mm)	Shoot	48.2^{ab}	43.8^{b}	52.2^{ab}	55.7^{a}
		Root*	70.6^{c}	72.0^{bc}	88.9^{a}	78.3^{b}
	FW (mg)	Shoot	288.7^{b}	162.9^{c}	342.0^{ab}	352.2^{a}
		Roots	264.8^{a}	208.1^{b}	288.6^{a}	275.1^{a}
		Cotyledons	503.8^{a}	369.2^{b}	528.2^{a}	476.4^{a}
	DW (mg)	Shoots	48.1^{ab}	43.3^{b}	47.9^{ab}	54.7^{a}
		Roots	20.8^{a}	17.3^{a}	22.6^{a}	22.4^{a}
		Cotyledons	153.4^{a}	129.0^{b}	136.0^{ab}	126.6^{b}

*- the length of the primary root

Table S4. The concentration of total identified polar metabolites (TIPMs), including total soluble carbohydrates (TSCs), total amino acids (TAAs), total organic acids (TOAs), and total remaining compounds (TRCs) **in shoots** of 21-day-old pea seedlings (*Pisum sativum* L.), developed from seeds after water and bio-AgNPs priming, 14 days post-inoculation with *D. pinodes* or without inoculation. Means of 3 replicates. The same letters by the values indicate statistically insignificant differences (p<0.05) based on two-way ANOVA analysis and Tukey's post-hoc test (valid in rows).

	Water priming		bio-AgNPs priming 100 mg/L for 1h	
	no infection	infection	no infection	infection
**Metabolites**	mg/gDW			
**TIPMs**, including:	157.49^{a}	121.96^{b}	128.93^{b}	123.73^{b}
**TSCs**, including:	51.49^{a}	34.53^{c}	39.34^{b}	29.39^{d}
fructose	0.83^{a}	0.49^{b}	0.58^{b}	0.54^{b}
galactitol	1.76^{bc}	1.80^{b}	2.13^{a}	1.56^{c}
glucose	6.33^{a}	2.91^{c}	5.28^{b}	5.62^{b}
maltose	0.23^{c}	0.78^{a}	0.13^{d}	0.60^{b}
mannitol	0.94^{b}	0.85^{b}	0.93^{b}	1.10^{a}
myo-inositol	2.97^{a}	2.23^{b}	2.10^{b}	2.01^{b}
raffinose	0.03^{a}	0.03^{a}	0.03^{a}	0.02^{a}
stachyose	0.20^{a}	0.23^{a}	0.17^{a}	0.20^{a}
sucrose	37.27^{a}	24.29^{b}	26.82^{b}	16.61^{c}
trehalose	0.48^{b}	0.91^{a}	0.45^{b}	0.64^{ab}
gluconic acid	0.69^{c}	0.78^{bc}	0.84^{b}	1.11^{a}
**TAAs**, including:	92.35^{a}	69.98^{b}	73.38^{b}	75.35^{b}
alanine	0.78^{a}	1.02^{a}	0.86^{a}	0.82^{a}
asparagine	33.52^{a}	16.75^{c}	28.15^{b}	27.25^{b}
aspartic acid	0.96^{c}	2.00^{a}	1.07^{c}	1.43^{b}
β-alanine	0.08^{b}	0.08^{b}	0.12^{ab}	0.15^{a}
GABA	1.15^{b}	1.80^{a}	1.24^{b}	1.64^{a}
glutamic acid	1.24^{a}	1.15^{a}	0.92^{a}	1.25^{a}
homoserine	43.62^{a}	36.27^{b}	32.43^{c}	35.05^{bc}
hydroxyproline	0.84^{c}	0.86^{c}	1.92^{a}	1.49^{b}
isoleucine	0.58^{a}	0.63^{a}	0.60^{a}	0.61^{a}
phenylalanine	0.50^{ab}	0.52^{a}	0.44^{b}	0.47^{ab}
proline	4.78^{a}	5.30^{a}	1.85^{b}	1.41^{b}
serine	1.08^{a}	0.88^{b}	0.90^{b}	0.87^{b}
threonine	1.45^{a}	0.97^{b}	0.97^{b}	1.03^{b}
valine	1.78^{a}	1.74^{a}	1.92^{a}	1.90^{a}
**TOAs**, including:	4.06^{b}	5.50^{a}	3.39^{c}	4.09^{b}
butyric acid	0.33^{b}	0.52^{a}	0.18^{c}	0.31^{b}
citric acid	1.36^{a}	1.28^{a}	0.98^{b}	1.20^{a}
lactic acid	0.14^{b}	0.56^{a}	0.06^{b}	0.14^{b}
malic acid	1.08^{b}	1.50^{a}	1.04^{b}	1.06^{b}
malonic acid	0.22^{b}	0.16^{c}	0.24^{a}	0.21^{b}
oxalic acid	0.20^{b}	0.25^{ab}	0.21^{b}	0.32^{a}
propionic acid	0.43^{a}	0.23^{b}	0.16^{c}	0.19^{b}
succinic acid	0.31^{d}	0.99^{a}	0.51^{c}	0.67^{b}
**TRCs**, including:	9.59^{c}	10.80^{bc}	11.52^{b}	13.45^{a}
phosphoric acid	9.35^{c}	10.63^{bc}	11.08^{b}	12.91^{a}
urea	0.24^{c}	0.17^{c}	0.45^{b}	0.54^{a}
UNK*	0.87^{b}	0.37^{c}	1.16^{a}	0.84^{b}

*Unknown soluble carbohydrate, not included to TSCs and TIPMs.

Table S5. The concentration of total identified polar metabolites (TIPMs), including total soluble carbohydrates (TSCs), total amino acids (TAAs), total organic acids (TOAs), and total remaining compounds (TRCs) **in roots** of 21-day-old pea seedlings (*Pisum sativum* L.), developed from seeds after water and bio-AgNPs priming, 14 days post-inoculation with *D. pinodes* or without inoculation. Means of 3 replicates. The same letters by the values indicate statistically insignificant differences (p<0.05) based on two-way ANOVA analysis and Tukey's post-hoc test (valid in rows).

	Water priming for 1 h		bio-AgNPs priming 100 mg/L for 1 h	
	no infection	infection	no infection	infection
**Metabolites**	mg/gDW			
**TIPMs**, including:	87.83^{a}	68.76^{b}	57.75^{c}	70.17^{b}
**TSCs**, including:	29.04^{a}	21.50^{b}	14.48^{c}	19.85^{b}
fructose	0.54^{a}	0.22^{b}	0.10^{c}	0.16^{bc}
galactitol	1.14^{ab}	0.71^{c}	1.01^{b}	1.24^{a}
glucose	2.92^{a}	2.33^{b}	2.79^{a}	2.95^{a}
maltose	1.00^{a}	1.17^{a}	0.17^{b}	0.26^{b}
mannitol	0.90^{a}	0.79^{ab}	0.73^{b}	0.86^{ab}
myo-inositol	2.30^{a}	1.69^{b}	1.27^{c}	1.75^{b}
stachyose	0.10^{a}	0.00^{d}	0.03^{c}	0.08^{b}
sucrose	20.44^{a}	15.05^{b}	7.87^{d}	12.07^{c}
trehalose	0.23^{b}	0.39^{a}	0.18^{b}	0.25^{ab}
gluconic acid	0.47^{a}	0.33^{b}	0.50^{a}	0.49^{a}
**TAAs**, including:	46.23^{a}	37.37^{c}	34.28^{d}	40.00^{b}
alanine	0.40^{a}	0.36^{a}	0.36^{a}	0.37^{a}
asparagine	5.98^{b}	5.49^{c}	4.69^{d}	6.51^{a}
aspartic acid	0.65^{a}	0.47^{b}	0.40^{c}	0.46^{b}
β-alanine	0.10^{a}	0.05^{a}	0.02^{a}	0.06^{a}
GABA	0.56^{b}	0.06^{d}	0.37^{c}	1.31^{a}
glutamic acid	0.20^{b}	0.35^{a}	0.26^{ab}	0.33^{a}
homoserine	36.22^{a}	28.00^{b}	26.41^{c}	28.92^{b}
hydroxyproline	0.35^{b}	0.21^{c}	0.41^{ab}	0.50^{a}
isoleucine	0.26^{a}	0.20^{b}	0.27^{a}	0.27^{a}
phenylalanine	0.18^{a}	0.15^{b}	0.18^{a}	0.19^{a}
proline	0.24^{b}	0.92^{a}	0.03^{c}	0.04^{c}
serine	0.51^{a}	0.43^{b}	0.30^{d}	0.32^{c}
threonine	0.26^{a}	0.38^{a}	0.34^{a}	0.37^{a}
valine	0.33^{a}	0.30^{b}	0.24^{c}	0.33^{a}
**TOAs**, including:	4.45^{a}	3.67^{b}	2.46^{c}	2.58^{c}
butyric acid	0.41^{a}	0.25^{ab}	0.08^{b}	0.12^{b}
citric acid	0.65^{a}	0.66^{a}	0.48^{c}	0.60^{b}
lactic acid	0.48^{a}	0.19^{c}	0.25^{b}	0.17^{c}
malic acid	1.29^{b}	1.49^{a}	1.01^{c}	1.04^{c}
malonic acid	0.11^{a}	0.11^{a}	0.08^{b}	0.08^{b}
oxalic acid	0.25^{a}	0.14^{b}	0.22^{a}	0.24^{a}
propionic acid	1.11^{a}	0.64^{b}	0.13^{c}	0.09^{c}
succinic acid	0.15^{d}	0.18^{c}	0.21^{b}	0.23^{a}
**TRCs**, including:	8.11^{a}	6.22^{c}	6.53^{c}	7.75^{b}
phosphoric acid	8.01^{a}	6.16^{c}	6.31^{c}	7.51^{b}
urea	0.10^{b}	0.07^{b}	0.22^{a}	0.23^{a}
UNK*	1.34^{c}	1.95^{b}	2.70^{a}	2.48^{a}

*Unknown soluble carbohydrate, not included to TSCs and TIPMs.

Table S6. The concentration of total identified polar metabolites (TIPMs), including total soluble carbohydrates (TSCs), total amino acids (TAAs), total organic acids (TOAs), and total remaining compounds (TRCs) **in cotyledons** of 21-day-old pea seedlings (*Pisum sativum* L.), developed from seeds after water and bio-AgNPs priming, 14 days post-inoculation with *D. pinodes* or without inoculation. Means of 3 replicates. The same letters by the values indicate statistically insignificant differences (p<0.05) based on two-way ANOVA analysis and Tukey's post-hoc test (valid in rows).

	Water priming for 1 h		bio-AgNPs priming 100 mg/L for 1 h	
	no infection	infection	no infection	infection
**Metabolites**	mg/gDW			
**TIPMs**, including:	175.42^{a}	177.59^{a}	181.68^{a}	154.68^{b}
**TSCs**, including:	125.25^{b}	127.10^{b}	142.50^{a}	118.98^{b}
fructose	2.09^{b}	2.74^{a}	0.67^{d}	1.17^{c}
galactitol	0.42^{a}	0.21^{b}	0.05^{c}	0.06^{c}
glucose	0.70^{a}	0.72^{a}	0.63^{a}	0.69^{a}
mannitol	0.14^{c}	0.13^{c}	0.30^{a}	0.24^{b}
myo-inositol	5.63	5.62	5.89	5.37
raffinose	0.11^{a}	0.13^{a}	0.10^{a}	0.12^{a}
stachyose	0.06^{b}	0.07^{b}	0.18^{a}	0.22^{a}
sucrose	115.02^{b}	116.22^{b}	133.70^{a}	110.45^{b}
trehalose	0.49^{a}	0.60^{a}	0.54^{a}	0.36^{a}
verbascose	0.03^{b}	0.03^{b}	0.13^{a}	0.14^{a}
erythronic acid	0.02^{c}	0.02^{c}	0.10^{a}	0.06^{b}
gluconic acid	0.53^{a}	0.61^{a}	0.22^{b}	0.12^{b}
**TAAs**, including	38.09^{a}	38.03^{a}	29.10^{b}	26.30^{b}
alanine	1.29^{a}	1.45^{a}	0.79^{b}	0.94^{b}
asparagine	9.90^{a}	8.37^{b}	7.91^{b}	7.63^{b}
aspartic acid	0.68^{b}	0.81^{a}	0.59^{b}	0.59^{b}
β-alanine	0.13^{a}	0.13^{a}	0.08^{b}	0.12^{a}
GABA	3.12^{b}	3.64^{a}	1.65^{d}	1.91^{c}
glutamic acid	0.25^{ab}	0.29^{a}	0.20^{b}	0.20^{b}
homoserine	10.47^{a}	9.57^{b}	7.35^{c}	5.28^{d}
hydroxyproline	0.37^{a}	0.38^{a}	0.34^{a}	0.31^{a}
isoleucine	1.96^{b}	2.38^{a}	1.49^{c}	1.60^{c}
lysine	0.61^{b}	0.51^{c}	0.51^{c}	0.73^{a}
phenylalanine	3.02^{a}	3.16^{a}	2.24^{b}	2.22^{b}
proline	0.45^{b}	0.92^{a}	0.10^{c}	0.16^{c}
serine	2.36^{a}	2.00^{b}	1.60^{c}	1.92^{b}
threonine	0.81^{a}	0.83^{a}	0.45^{b}	0.57^{b}
tryptophan	1.92^{a}	2.46^{a}	2.80^{a}	1.22^{a}
valine	0.77^{b}	1.12^{a}	0.99^{ab}	0.90^{ab}
**TOAs**, including:	4.89^{b}	6.19^{a}	3.68^{c}	3.86^{c}
butyric acid	0.03^{ab}	0.05^{a}	0.02^{b}	0.02^{b}
citric acid	1.42^{ab}	1.42^{a}	1.18^{c}	1.23^{bc}
lactic acid	0.19^{b}	0.50^{a}	0.03^{b}	0.05^{b}
malic acid	0.44^{a}	0.60^{a}	0.54^{a}	0.34^{a}
malonic acid	0.03^{b}	0.06^{a}	0.05^{a}	0.05^{a}
oxalic acid	1.57^{ab}	1.58^{a}	1.31^{c}	1.36^{bc}
propionic acid	0.17^{a}	0.11^{b}	0.05^{c}	0.06^{c}
succinic acid	1.03^{b}	1.87^{a}	0.50^{d}	0.76^{c}
**TRCs**, including:	7.19^{a}	6.27^{ab}	6.39^{ab}	5.54^{b}
phosphoric acid	7.08^{a}	5.99^{b}	6.35^{ab}	5.49^{b}
urea	0.11^{ab}	0.28^{a}	0.05^{b}	0.04^{b}
UNK*	0.36^{bc}	0.53^{a}	0.33^{c}	0.38^{b}

*Unknown soluble carbohydrate, not included to TSCs and TIPMs.

Table S7. The concentration of total identified polar metabolites (TIPMs), including total soluble carbohydrates (TSCs), total amino acids (TAAs), total organic acids (TOAs), and total remaining compounds (TRCs) **in shoots** of 21-day-old pea seedlings (*Pisum sativum* L.), developed from seeds after water and bio-AgNPs priming, 14 days post-inoculation with *F. avenaceum* or without inoculation. Means of 3 replicates. The same letters by the values indicate statistically insignificant differences (*p*<0.05) based on two-way ANOVA analysis and Tukey's post-hoc test (valid in rows).

	Water priming for 1 h		bio-AgNPs priming 100 mg/L for 1 h	
	no infection	infection	no infection	infection
**Metabolites**	mg/gDW			
**TIPMs**, including:	157.49[a]	158.11[a]	128.93[b]	129.69[b]
**TSCs**, including:	51.49[a]	50.76[a]	39.34[b]	54.10[a]
fructose	0.83[a]	0.42[b]	0.58[b]	0.93[a]
galactitol	1.76[b]	1.73[b]	2.13[a]	1.69[b]
glucose	6.33[b]	3.34[d]	5.28[c]	6.87[a]
maltose	0.23[b]	0.84[a]	0.13[b]	0.34[b]
mannitol	0.94[b]	0.82[c]	0.93[b]	1.01[a]
myo-inositol	2.97[a]	1.90[c]	2.10[c]	2.55[b]
raffinose	0.03[a]	0.05[a]	0.03[a]	0.03[a]
stachyose	0.20[a]	0.22[a]	0.17[a]	0.20[a]
sucrose	37.27[a]	40.13[a]	26.82[b]	39.34[a]
trehalose	0.48[b]	1.20[a]	0.45[b]	0.52[b]
gluconic acid	0.69[c]	0.96[a]	0.84[b]	0.96[a]
**TAAs**, including:	92.35[a]	88.97[a]	73.38[b]	62.36[c]
alanine	0.78[bc]	0.96[a]	0.86[ab]	0.65[c]
asparagine	33.52[a]	23.82[c]	28.15[b]	21.97[d]
aspartic acid	0.96[b]	1.67[a]	1.07[b]	0.80[c]
β-alanine	0.08[b]	0.12[a]	0.12[a]	0.07[b]
GABA	1.15[a]	1.19[a]	1.24[a]	1.23[a]
glutamic acid	1.24[ab]	1.76[a]	0.92[b]	0.88[b]
homoserine	43.62[b]	47.11[a]	32.43[c]	30.19[d]
hydroxyproline	0.84[b]	0.66[b]	1.92[a]	1.15[b]
isoleucine	0.58[a]	0.61[a]	0.60[a]	0.50[b]
phenylalanine	0.50[b]	0.63[a]	0.44[c]	0.32[d]
proline	4.78[b]	6.66[a]	1.85[c]	1.33[c]
serine	1.08[a]	0.99[ab]	0.90[b]	0.89[b]
threonine	1.45[a]	1.19[b]	0.97[bc]	0.81[c]
valine	1.78[b]	1.59[c]	1.92[a]	1.57[c]
**TOAs**, including:	4.06[b]	6.45[a]	3.39[c]	2.87[d]
butyric acid	0.33[b]	1.24[a]	0.18[c]	0.16[c]
citric acid	1.36[b]	1.54[a]	0.98[c]	0.85[c]
lactic acid	0.14[b]	0.33[a]	0.06[b]	0.09[b]
malic acid	1.08[b]	1.80[a]	1.04[b]	0.95[c]
malonic acid	0.22[b]	0.23[ab]	0.24[a]	0.13[c]
oxalic acid	0.20[b]	0.29[a]	0.21[b]	0.21[b]
propionic acid	0.43[a]	0.28[b]	0.16[c]	0.16[c]
succinic acid	0.31[c]	0.73[a]	0.51[b]	0.32[c]
**TRCs**, including:	9.59[c]	10.84[b]	11.52[a]	9.04[d]
phosphoric acid	9.35[b]	10.68[a]	11.08[a]	8.77[c]
urea	0.24[b]	0.16[c]	0.45[a]	0.27[b]
UNK*	0.87[c]	0.26[d]	1.16[a]	0.98[b]

*Unknown soluble carbohydrate, not included to TSCs and TIPMs.

Table S8. The concentration of total identified polar metabolites (TIPMs), including total soluble carbohydrates (TSCs), total amino acids (TAAs), total organic acids (TOAs), and total remaining compounds (TRCs) **in roots** of 21-day-old pea seedlings (*Pisum sativum* L.), developed from seeds after water and bio-AgNPs priming, 14 days post-inoculation with *F. avenaceum* or without inoculation. Means of 3 replicates. The same letters by the values indicate statistically insignificant differences (p<0.05) based on two-way ANOVA analysis and Tukey's post-hoc test (valid in rows).

	Water priming for 1 h		bio-AgNPs priming 100 mg/L for 1 h	
	no infection	infection	no infection	infection
**Metabolites**	mg/gDW			
**TIPMs**, including:	87.83^{a}	90.35^{a}	57.75^{c}	66.88^{b}
**TSCs**, including:	29.04^{b}	33.48^{a}	14.48^{d}	20.01^{c}
fructose	0.54^{a}	0.38^{b}	0.10^{c}	0.14^{c}
galactitol	1.14^{a}	0.68^{b}	1.01^{a}	1.08^{a}
glucose	2.92^{a}	2.47^{b}	2.79^{ab}	3.00^{a}
maltose	1.00^{a}	0.68^{b}	0.17^{c}	0.22^{c}
mannitol	0.90^{b}	0.79^{c}	0.73^{c}	1.01^{a}
myo-inositol	2.30^{a}	1.07^{d}	1.27^{c}	1.66^{b}
stachyose	0.10^{a}	0.10^{a}	0.03^{b}	0.10^{a}
sucrose	20.44^{b}	27.10^{a}	7.87^{d}	12.24^{c}
trehalose	0.23^{b}	0.54^{a}	0.18^{b}	0.20^{b}
gluconic acid	0.47^{a}	0.35^{b}	0.50^{a}	0.58^{a}
**TAAs**, including:	46.23^{a}	44.96^{a}	34.28^{b}	37.12^{b}
alanine	0.40^{a}	0.41^{a}	0.36^{a}	0.43^{a}
asparagine	5.98^{a}	3.45^{b}	4.69^{b}	4.45^{b}
aspartic acid	0.65^{a}	0.46^{b}	0.40^{b}	0.45^{b}
β-alanine	0.10^{a}	0.02^{a}	0.02^{a}	0.05^{a}
GABA	0.56^{b}	0.19^{c}	0.37^{bc}	1.34^{a}
glutamic acid	0.20^{a}	0.23^{a}	0.26^{a}	0.22^{a}
homoserine	36.22^{a}	35.21^{a}	26.41^{b}	28.31^{b}
hydroxyproline	0.35^{a}	0.39^{a}	0.41^{a}	0.38^{a}
isoleucine	0.26^{b}	0.26^{ab}	0.27^{ab}	0.28^{a}
phenylalanine	0.18^{a}	0.12^{b}	0.18^{a}	0.15^{a}
proline	0.24^{b}	3.27^{a}	0.03^{c}	0.03^{bc}
serine	0.51^{a}	0.54^{a}	0.30^{b}	0.37^{b}
threonine	0.26^{a}	0.44^{a}	0.34^{a}	0.37^{a}
valine	0.33^{b}	0.39^{a}	0.24^{c}	0.30^{b}
**TOAs**, including:	4.45^{a}	4.43^{a}	2.46^{b}	2.49^{b}
butyric acid	0.41^{a}	0.35^{ab}	0.08^{c}	0.12^{bc}
citric acid	0.65^{a}	0.56^{b}	0.48^{bc}	0.45^{c}
lactic acid	0.48^{b}	0.67^{a}	0.25^{c}	0.29^{c}
malic acid	1.29^{ab}	1.53^{a}	1.01^{b}	0.99^{b}
malonic acid	0.11^{a}	0.11^{a}	0.08^{b}	0.07^{c}
oxalic acid	0.25^{ab}	0.15^{c}	0.22^{b}	0.27^{a}
propionic acid	1.11^{a}	0.89^{b}	0.13^{c}	0.11^{c}
succinic acid	0.15^{b}	0.19^{a}	0.21^{a}	0.20^{a}
**TRCs**, including:	8.11^{a}	7.48^{b}	6.53^{c}	7.26^{b}
phosphoric acid	8.01^{a}	7.35^{b}	6.31^{c}	7.13^{b}
urea	0.10^{b}	0.13^{b}	0.22^{a}	0.13^{b}
UNK*	1.34^{c}	1.32^{c}	2.70^{a}	2.46^{b}

*Unknown soluble carbohydrate, not included to TSCs and TIPMs.

Table S9. The concentration of total identified polar metabolites (TIPMs), including total soluble carbohydrates (TSCs), total amino acids (TAAs), total organic acids (TOAs), and total remaining compounds (TRCs) **in cotyledons** of 21-day-old pea seedlings (*Pisum sativum* L.), developed from seeds after water and bio-AgNPs priming, 14 days post-inoculation with *F. avenaceum* or without inoculation. Means of 3 replicates. The same letters by the values indicate statistically insignificant differences ($p<0.05$) based on two-way ANOVA analysis and Tukey's post-hoc test (valid in rows).

	Water priming for 1 h		bio-AgNPs priming 100 mg/L for 1 h	
	no infection	infection	no infection	infection
**Metabolites**	mg/gDW			
**TIPMs**, including:	175.42^{bc}	194.29^{a}	181.68^{b}	169.16^{c}
**TSCs**, including:	125.25^{c}	150.72^{a}	142.50^{b}	137.50^{b}
fructose	2.09^{b}	2.58^{a}	0.67^{d}	1.32^{c}
galactitol	0.42^{b}	0.55^{a}	0.05^{c}	0.08^{c}
glucose	0.70^{b}	1.04^{ab}	0.63^{b}	1.19^{a}
mannitol	0.14^{d}	0.22^{c}	0.30^{b}	0.56^{a}
myo-inositol	5.63^{bc}	6.01^{a}	5.89^{ab}	5.52^{c}
raffinose	0.11^{b}	0.14^{a}	0.10^{bc}	0.09^{c}
stachyose	0.06^{c}	0.07^{c}	0.18^{a}	0.16^{b}
sucrose	115.02^{c}	138.64^{a}	133.70^{ab}	127.76^{b}
trehalose	0.49^{a}	0.71^{a}	0.54^{a}	0.49^{a}
verbascose	0.03^{c}	0.02^{c}	0.13^{a}	0.10^{b}
erythronic acid	0.02^{b}	0.02^{b}	0.10^{a}	0.09^{a}
gluconic acid	0.53^{b}	0.72^{a}	0.22^{c}	0.14^{c}
**TAAs**, including	38.09^{a}	34.04^{ab}	29.10^{b}	22.57^{c}
alanine	1.29^{a}	1.04^{b}	0.79^{c}	0.71^{c}
asparagine	9.90^{a}	8.80^{ab}	7.91^{b}	5.69^{c}
aspartic acid	0.68^{a}	0.53^{b}	0.59^{ab}	0.49^{b}
β-alanine	0.13^{a}	0.09^{b}	0.08^{b}	0.07^{b}
GABA	3.12^{a}	2.69^{b}	1.65^{c}	1.53^{c}
glutamic acid	0.25^{a}	0.14^{a}	0.20^{a}	0.22^{a}
homoserine	10.47^{a}	9.33^{b}	7.35^{c}	6.08^{d}
hydroxyproline	0.37^{a}	0.43^{a}	0.34^{a}	0.30^{a}
isoleucine	1.96^{a}	2.03^{a}	1.49^{b}	1.39^{b}
lysine	0.61^{a}	0.46^{a}	0.51^{a}	0.44^{a}
phenylalanine	3.02^{a}	2.75^{b}	2.24^{c}	1.90^{d}
proline	0.45^{b}	0.71^{a}	0.10^{c}	0.16^{c}
serine	2.36^{a}	2.10^{b}	1.60^{c}	1.38^{d}
threonine	0.81^{a}	0.74^{a}	0.45^{b}	0.44^{b}
tryptophan	1.92^{a}	1.57^{a}	2.80^{a}	0.99^{a}
valine	0.77^{b}	0.62^{b}	0.99^{a}	0.78^{b}
**TOAs**, including:	4.89^{a}	3.67^{b}	3.68^{b}	3.59^{b}
butyric acid	0.03^{b}	0.05^{a}	0.02^{c}	0.02^{bc}
citric acid	1.42^{a}	0.81^{b}	1.18^{ab}	1.19^{b}
lactic acid	0.19^{a}	0.05^{b}	0.03^{b}	0.04^{b}
malic acid	0.44^{b}	0.55^{a}	0.54^{a}	0.42^{b}
malonic acid	0.03^{b}	0.04^{b}	0.05^{a}	0.04^{b}
oxalic acid	1.57^{a}	0.89^{b}	1.31^{ab}	1.32^{a}
propionic acid	0.17^{a}	0.24^{a}	0.05^{b}	0.05^{b}
succinic acid	1.03^{a}	1.03^{a}	0.50^{b}	0.50^{b}
**TRCs**, including:	7.19^{a}	5.86^{b}	6.39^{ab}	5.50^{b}
phosphoric acid	7.08^{a}	5.83^{b}	6.35^{ab}	5.47^{b}
urea	0.11^{a}	0.02^{a}	0.05^{a}	0.03^{a}
UNK*	0.36^{ab}	0.40^{a}	0.33^{ab}	0.28^{b}

*Unknown soluble carbohydrate, not included to TSCs and TIPMs.

Figures

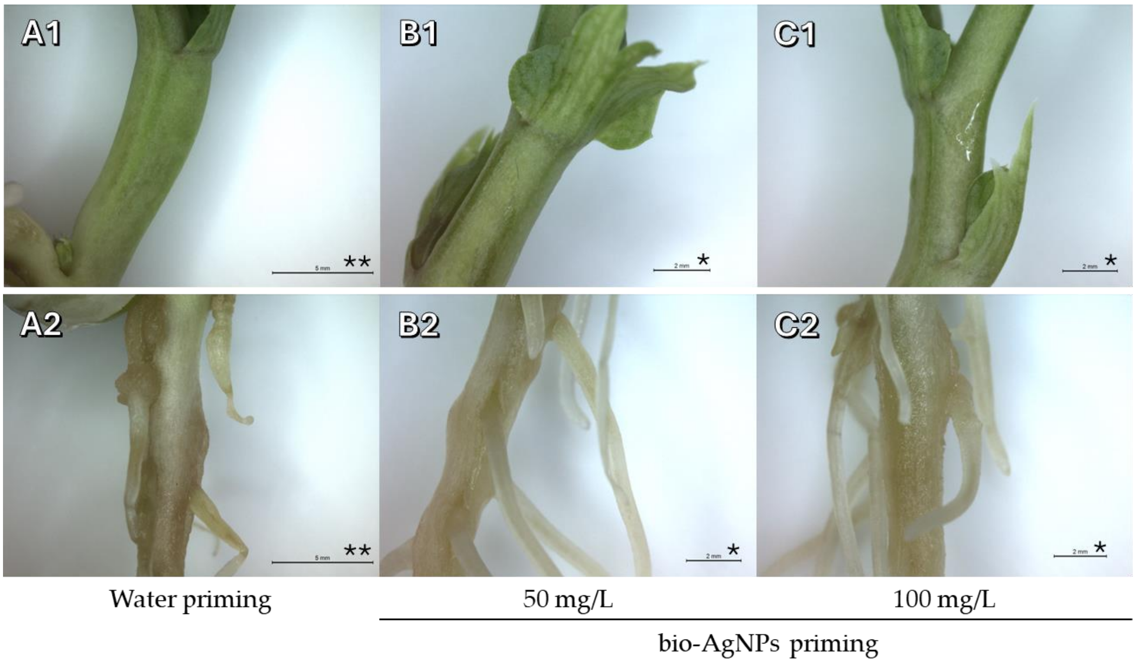

Figure S1. The base of the shoot and roots (**A1-C1** and **A2-C2**, respectively) of non-infected 21-day-old pea seedlings developed from seeds primed with water (**A1**, **A2**) and with bio-AgNPs at concentrations of 50 mg/L (**B1**, **B2)** and 100 mg/L (**C1**, **C2**). Scale bars: * = 2 mm and ** = 5 mm.

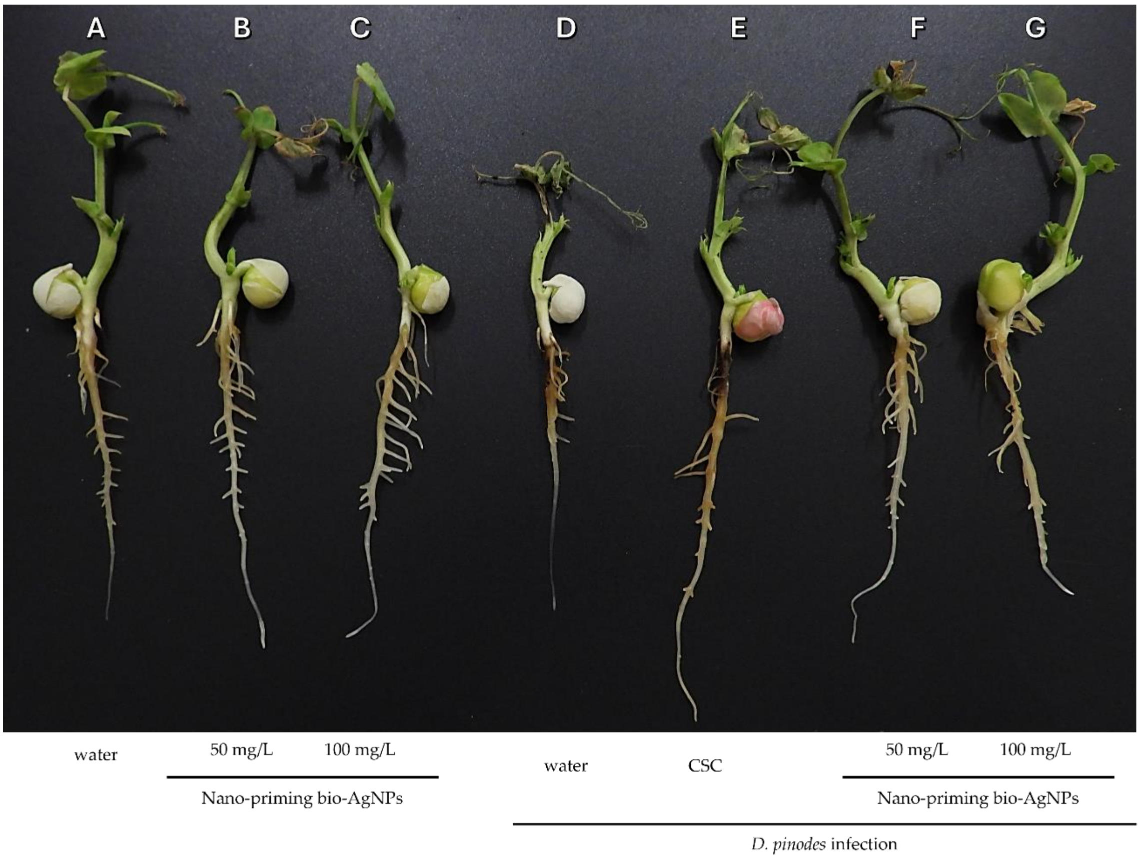

Figure S2. The 21-day-old pea seedlings not infected developed from seeds primed with water (**A**), bio-AgNPs at concentrations of 50 mg/L (**B)** and 100 mg/L (**C**) and seedlings 14 days after inoculation with *D. pinodes*, grown from seeds primed with water (**D**), commercial seed coat (CSC) mixture (Maxim 025 FS with 2.38% fludioxonil, 25 g/L; **E**) and bio-AgNPs at concentrations of 50 mg/L (**F)** and 100 mg/L (**G**).

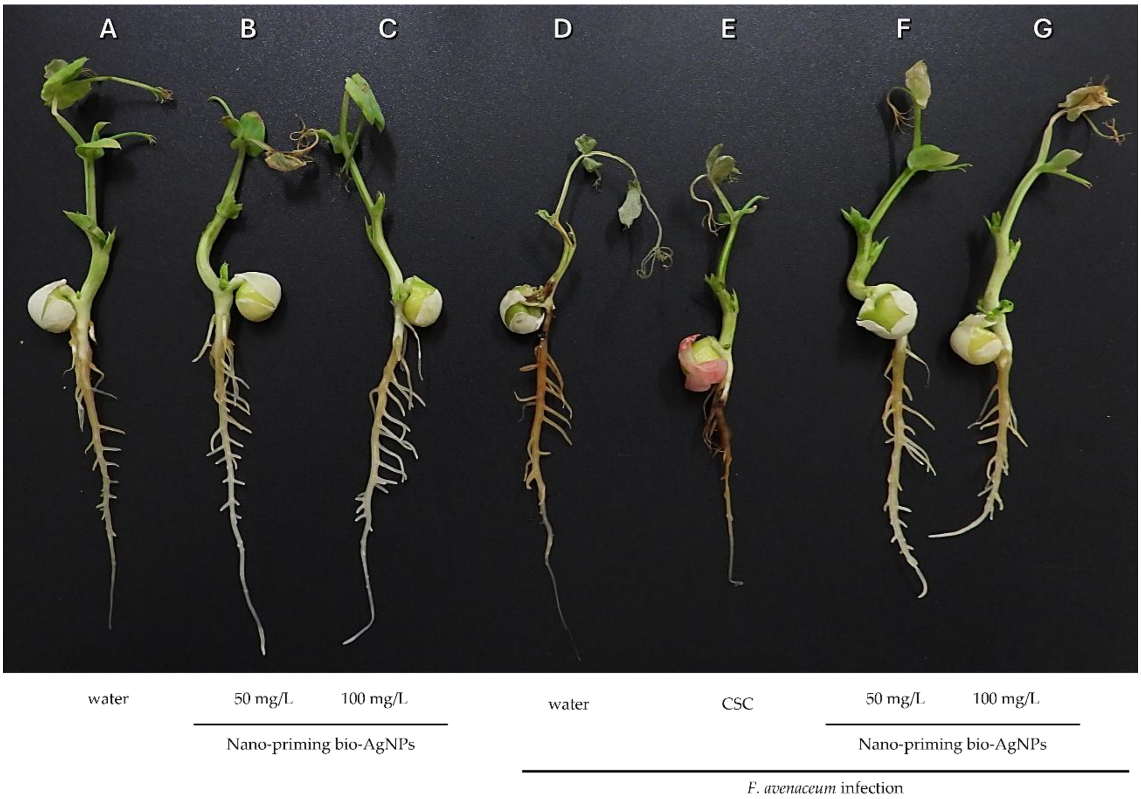

Figure S3. The 21-day-old pea seedlings not infected developed from seeds primed with water (**A**), bio-AgNPs at concentrations of 50 mg/L (**B)** and 100 mg/L (**C**) and seedlings 14 days after inoculation with *F. avenaceum*, grown from seeds primed with water (**D**), commercial seed coat (CSC) mixture (Maxim 025 FS with 2.38% fludioxonil, 25 g/L; **E**) and bio-AgNPs at concentrations of 50 mg/L (**F)** and 100 mg/L (**G**).

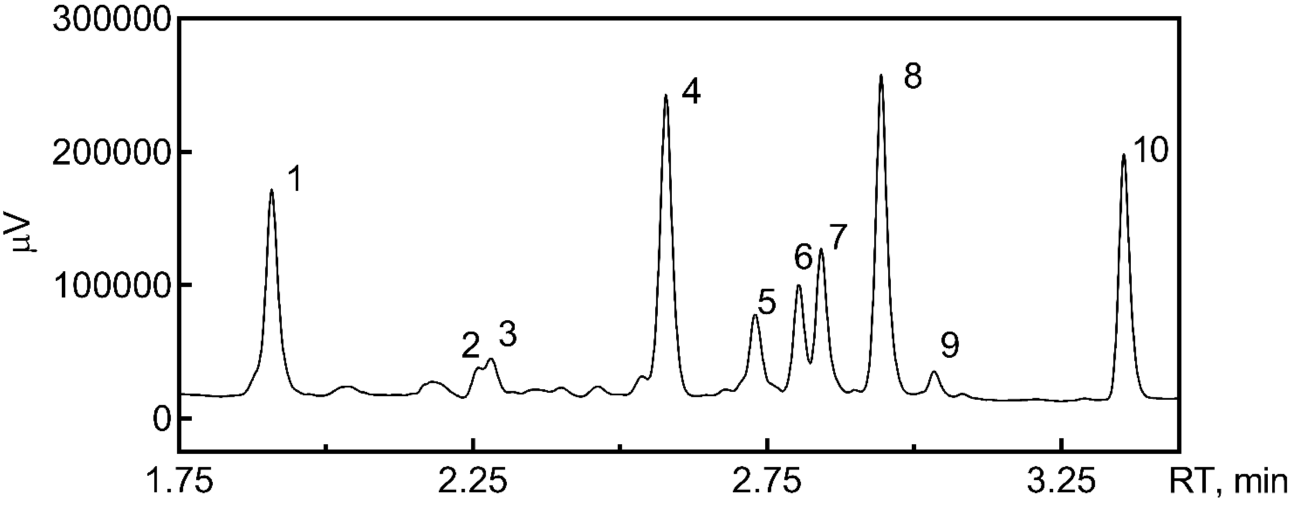

Figure S4. The fragment of the GC chromatogram of TMS-derivatives of oligosaccharides extracted from shoots of 21-day-old pea seedling developed from water-primed seeds. Notations: 1 – xylitol, internal standard; 2 and 3 – fructose; 4 and 8 – glucose; 5 – unknown; 6 – mannitol; 7 – galactitol; 9 – unknown; 10 – *myo*-inositol.

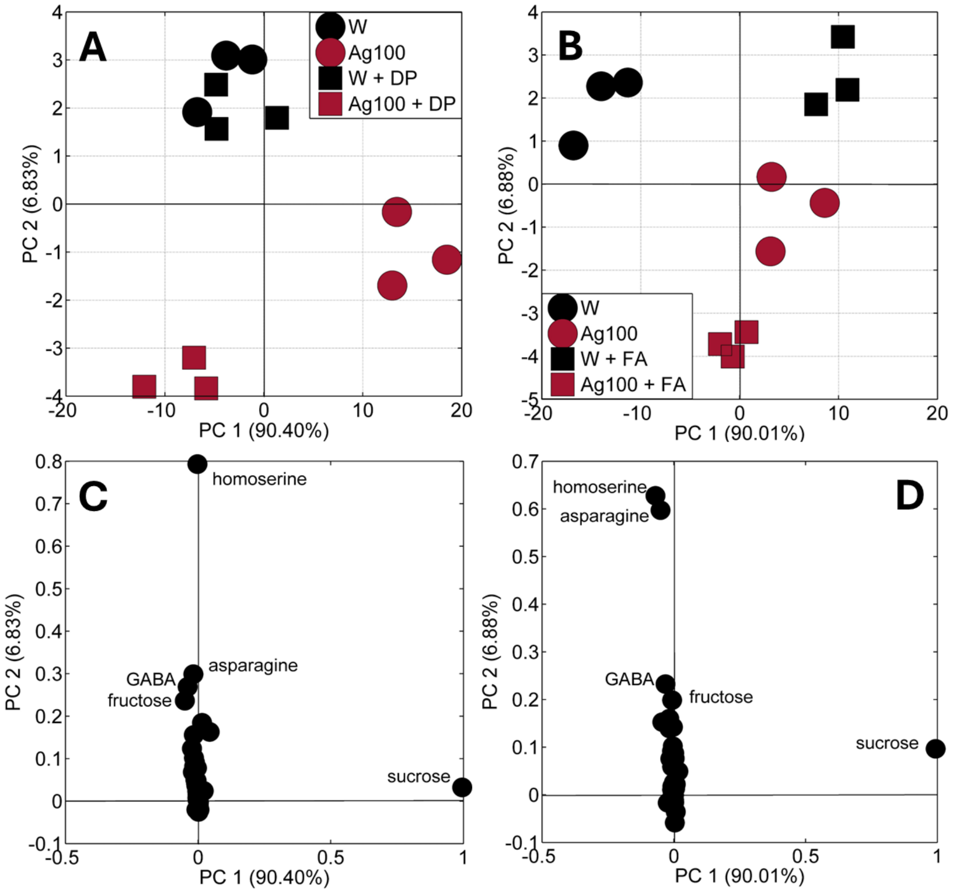

Figure S5. PCA score (**A**, **B**) and loading plots (**C**, **D**) of the metabolic profiles of the cotyledons of 21-day-old seedlings of pea (*Pisum sativum* L.) developed from seeds after water and bio-AgNPs priming, 14 days after *D. pinodes* (**A**, **C**) or *F. avenaceum* (**B**, **D**) inoculation. Abbreviations: W and W + DP/FA —non-infected seedlings and seedlings infected with *D. pinodes*/*F. avenaceum*, respectively, grown from water-primed seeds; Ag100 and Ag100 + DP/FA — non-infected seedlings and seedlings infected with *D. pinodes*/*F. avenaceum*, respectively, grown from seeds primed with bio-AgNPs at concentrations of 100 mg/L.

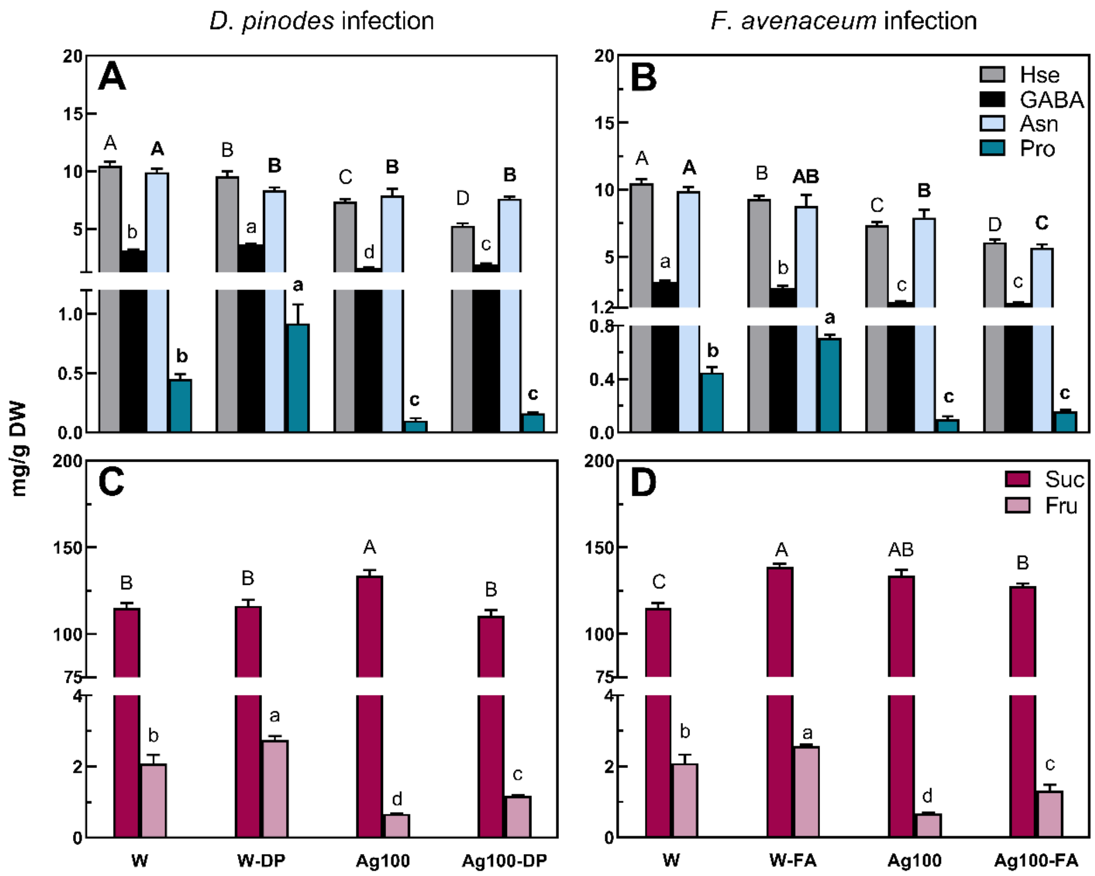

Figure S6. The concentrations of homoserine (Hse), γ-aminobutyric acid (GABA), asparagine (Asn), proline (Pro), sucrose (Suc) and fructose (Fru) in the cotyledons of 21-day-old seedlings of pea (*Pisum sativum* L.), developed for 21 days (from water- or bio-AgNPs-primed seeds) without infection or 14 days after inoculation of 7-day-old seedlings with *D. pinodes* (**A, C**) and *F. avenaceum* (**B, D**). Values (in mg/g DW) are the means of 3 replicates + SD. The same letters (A–D; a–d; **A–C**; **a–c**; separately for each metabolite) above the bars indicate statistically insignificant ($p < 0.05$) differences based on two-way ANOVA and Tukey's post–hoc test. Abbreviations: W and W-DP/FA—non-infected seedlings and seedlings infected with *D. pinodes*/*F. avenaceum*, respectively, grown from water-primed seeds; Ag100 and Ag100-DP/FA — non-infected seedlings and seedlings infected with *D. pinodes*/*F. avenaceum*, respectively, grown from seeds primed with bio-AgNPs at concentrations of 100 mg/L.
